# Morphological, Histological and Ultrastructural Characterization of the Common Dolphin’s Adrenal Glands

**DOI:** 10.3390/vetsci13040348

**Published:** 2026-04-02

**Authors:** Paula Alonso-Almorox, Alfonso Blanco, Ignacio Molpeceres-Diego, Raiden Grandía-Guzmán, Diego Llinás Rueda, Manuel Arbelo, Antonio Fernández

**Affiliations:** 1 Veterinary Histology and Pathology, Atlantic Center for Cetacean Research (CAIC), Institute of Animal Health and Food Safety (IUSA), Veterinary School, University of Las Palmas de Gran Canaria (ULPGC), Trasmontaña s/n, 35413 Arucas, Spainraiden.grandia@gmail.com (R.G.-G.); manuel.arbelo@ulpgc.es (M.A.); 2 Department of Anatomy and Comparative Pathology and Anatomy, University of Cordoba, 14014 Cordoba, Spain

**Keywords:** *Delphinus delphis*, cetaceans, adrenal gland, HPA axis, adrenal morphology, ultrastructure, stress physiology, neuroendocrine system

## Abstract

The adrenal glands are central regulators of endocrine function and stress physiology, yet detailed species-specific anatomical baselines remain limited in cetaceans. This study provides a comprehensive gross, histological, morphometric, and ultrastructural characterization of the adrenal glands in 55 short-beaked common dolphins (Delphinus delphis) examined postmortem in the Canary Islands. Adrenal glands were evaluated macroscopically and microscopically, and histological corticomedullary ratios were calculated from mid-transverse sections. Associations with body length, sexual maturity, and cause-of-death category were assessed statistically. Transmission electron microscopy was used to characterize cortical and medullary cellular ultrastructure. Adrenal weight showed a positive correlation with body length. The histological corticomedullary ratio showed no lateral asymmetry but differed significantly between sexually immature and mature individuals, indicating ontogenetic remodeling of adrenal architecture. In contrast, the corticomedullary ratio did not differ significantly between adult dolphins that died from acute events and those that died following more progressive pathological conditions. Ultrastructural analysis identified characteristic steroidogenic cortical cells and two distinct chromaffin cell populations in the medulla. These findings establish the first integrated anatomical baseline for the adrenal gland in Delphinus delphis, providing essential reference data for comparative anatomy, veterinary pathology, and the interpretation of endocrine-related findings in cetaceans.

## Simple Summary

The adrenal glands are small organs located near the kidneys that help regulate stress responses, metabolism, and many essential body functions. In dolphins and other marine mammals, hormone levels from these glands are often used to evaluate health and environmental stress. However, interpreting these signals correctly requires a clear understanding of what a healthy adrenal gland looks like in each species. For the short-beaked common dolphin, this detailed information has been lacking. In this study, we examined the adrenal glands of 55 short-beaked common dolphins (*Delphinus delphis*) stranded in the Canary Islands. We analysed their overall shape, internal structure, and microscopic organization. We found that the glands follow the typical mammalian pattern but also exhibit some species-specific structural features. The proportion between the outer and inner regions of the gland changed during development from immature to adult animals but did not differ between dolphins that died suddenly and those that died after prolonged illnesses. These results provide the first comprehensive structural reference for the adrenal gland in this species. This information will improve the interpretation of health assessments in stranded dolphins and support research on marine ecosystem health.

## 1. Introduction

The adrenal glands are key endocrine organs involved in the regulation of multiple physiological processes essential for mammalian homeostasis, including metabolism, cardiovascular function, immune modulation, and behavioral adaptation [1]. Through the synthesis and release of steroid hormones and catecholamines, the adrenal glands play a central role in both basal regulation and adaptive responses to internal and external challenges [2]. These functions are tightly integrated within the neuroendocrine system through the hypothalamic–pituitary–adrenal (HPA) axis, which coordinates central and peripheral signaling pathways to regulate adrenal activity and mediate the stress response [3,4,5,6]. As a result, adrenal hormones are widely used as biological indicators in clinical and veterinary contexts, particularly for assessing endocrine function and stress-related processes [7,8,9].

In cetaceans, which are increasingly recognized as sentinel species of marine ecosystem health and are exposed to diverse natural and anthropogenic stressors, the interpretation of adrenal-derived biomarkers has gained relevance [10,11,12,13]. However, meaningful interpretation of endocrine signals and biomarkers requires a thorough understanding of the underlying anatomical and cellular organization of the adrenal glands. Establishing detailed morphological and ultrastructural baselines is therefore a critical prerequisite for advancing physiological and pathological assessments in these species.

Structurally, the adrenal glands are paired organs composed of two embryologically and functionally distinct compartments: the cortex and the medulla [14]. The cortex is typically organized into three concentric zones (*zona glomerulosa*, *zona fasciculata*, and *zona reticularis*) distinguished by cellular arrangement, cytoplasmic composition, and endocrine output [15,16]. The medulla, derived from the neural crest, consists of chromaffin cells, arranged in cords or clusters within a dense sinusoidal network and is responsible for catecholamine synthesis and release [17]. Across mammals, variations in cortical zonation, capsular organization, trabecular architecture, and cortico-medullary relationships highlight the importance of detailed anatomical descriptions for identifying both conserved and species-specific traits [18,19,20].

In cetaceans, studies have documented a combination of conserved mammalian organization and recurrent species-specific features, with notable interspecific variability [21,22,23,24,25,26,27]. Among delphinids, cortical pseudolobulation associated with a thick capsule and prominent connective tissue septa is one of the most consistently reported characteristics [28]. Additional features include a distinct connective tissue layer separating cortex and medulla, and the presence of two chromaffin cell populations with differential staining properties, often interpreted as epinephrine- and norepinephrine-secreting cells [24,25,26]. These populations are frequently arranged as an outer, more intensely stained medullary band surrounding a lighter inner medulla.

Other reported traits include medullary protrusions extending through the cortex toward the capsule, cortical invaginations into the medulla around central vessels, and the presence of cell aggregates within the capsule interpreted as accessory adrenal tissue [24,25,26,27]. Alongside these qualitative features, morphometric parameters such as cortex-to-medulla (CM) ratios have been used to assess interspecific and intraspecific variation [10,26,29]. Despite these contributions, comprehensive species-specific studies integrating gross morphology, histology, and quantitative morphometry remain limited, constraining comparative and pathological interpretation in cetacean adrenal anatomy.

At the cellular level, ultrastructural analysis provides critical insight into adrenal organization, revealing features associated with steroidogenesis, catecholamine synthesis, and cell–vascular interactions. While transmission electron microscopy (TEM) has been widely applied in terrestrial mammals, its use in cetaceans remains scarce. Available studies are largely restricted to the medulla of the bottlenose dolphin (*Tursiops truncatus*), where distinct chromaffin cell populations have been described based on differences in granule morphology and cytoplasmic electron density [25]. However, ultrastructural characterization of the adrenal cortex, as well as integrative analyses encompassing both cortical and medullary compartments within a single species, remain largely unexplored.

Given the limited availability of integrative ultrastructural data in delphinids and the need for species-specific anatomical baselines, attention to widely distributed and frequently examined species is warranted. The short-beaked common dolphin (*Delphinus delphis*) is one of the most abundant odontocetes in temperate and subtropical waters and is commonly included in stranding and necropsy programs [30,31]. Despite its ecological relevance and frequent inclusion in stranding and necropsy programs, comprehensive morphological and ultrastructural characterizations of its adrenal glands remain lacking. In parallel with the increasing attention directed toward the stress response in marine mammals, characterization of the cetacean neuroendocrine system, particularly in relation to HPA axis regulation and stress physiology, has become progressively more relevant [32,33,34]. Establishing a detailed anatomical baseline for this species is therefore essential to support both comparative studies within Delphinidae and the interpretation of pathological alterations.

The aim of the present study is to provide an integrative morphological, histological, morphometric, and ultrastructural characterization of the adrenal glands in the short-beaked common dolphin. By combining multiple levels of anatomical analysis, this work establishes a comprehensive reference framework for the species and contributes baseline data relevant for veterinary histopathology, comparative anatomy, and the study of the cetacean neuroendocrine system.

## 2. Materials and Methods

### 2.1. Animals

Adrenal glands were collected from 55 short-beaked common dolphins (*Delphinus delphis*) examined postmortem. All animals underwent complete post-mortem examinations performed by veterinarians from the pathology team at the Animal Health and Food Safety Institute (IUSA), Faculty of Veterinary Medicine, Universidad de Las Palmas de Gran Canaria (ULPGC), following the standardized cetacean necropsy protocol described by Kuiken and García Hartmann, with modifications routinely applied by the IUSA pathology team [35].

To minimize the influence of post-mortem autolysis and terminal pathological processes, only specimens classified as fresh or very fresh and showing no advanced tissue degradation were included in the histological, morphometric, and ultrastructural analyses [36]. Particular attention was given to the preservation of tissue architecture and cellular detail during sample selection. Additionally, animals showing major adrenal pathological alterations (e.g., extensive necrosis, hemodynamic changes, neoplasia, or severe inflammatory lesions) were excluded. This approach was adopted to ensure that the present study reflects baseline morphological and ultrastructural characteristics of the species.

For each individual, sex, total body length, and sexual maturity status (determined by gonadal histology) were recorded. The cause of death was established based on gross and histopathological findings (e.g., infectious disease, parasitic disease, fishing interaction, degenerative processes). The complete morphometric and histomorphometric dataset for all examined individuals is provided in Supplementary Table S1.

Sampling of stranded dolphins from the Canary Islands was authorized by the Spanish Ministry for the Ecological Transition and Demographic Challenge and the Canarian Government’s Environmental Department (project number PID2021-127687NB-10). A copy of the permit is provided in the Supplementary Materials.

### 2.2. Adrenal Gland Collection and Sampling

During necropsy, adrenal glands were identified and dissected in situ. Both glands were excised by sharp dissection and surrounding adipose and connective tissues were carefully removed to expose the gland surface without compromising the capsule.

Prior to fixation, each gland was weighed using a precision balance and measured macroscopically to obtain length and maximum width. Following gross examination, the glands were sectioned transversely through their longitudinal axis. A central transverse section was used to assess internal architecture and cortico–medullary organization. Subsequently, tissue samples approximately 0.5 cm in thickness were obtained from the central transverse plane for histological and ultrastructural processing.

### 2.3. Histochemical Study

For histological and histochemical analysis, adrenal tissue samples were fixed by immersion in 10% neutral-buffered formalin, routinely processed, and embedded in paraffin. Serial sections of 5 μm thickness were obtained.

A panel of classical histochemical stains was selected to characterize complementary structural and functional aspects of the adrenal gland. Hematoxylin and eosin (H&E) was used for general tissue architecture and cellular morphology. Masson’s trichrome staining was applied to assess connective tissue organization, including capsular and trabecular components. Periodic acid–Schiff (PAS) and PAS with diastase digestion (PAS-D) were used to evaluate carbohydrate-rich structures and to differentiate glycogen content from other PAS-positive substances. Grimelius silver staining was employed to identify argyrophilic cells, particularly within the adrenal medulla, facilitating the characterization of chromaffin cell populations based on their staining properties.

Histological evaluation and image acquisition were performed using an Olympus BX51 light microscope equipped with a DP21 digital camera and a 0.5× adapter (Olympus Corp., Tokyo, Japan). The microscope’s integrated measurement software was used for quantitative assessment of histological features.

### 2.4. Morphometric Analysis

Morphometric variables were examined in relation to biological parameters including sex, total body length, and sexual maturity (determined based on gonadal histological evaluation).

Quantitative morphometric analyses were conducted at both the macroscopic (gross anatomical) and histological levels.

At necropsy, macroscopic measurements included adrenal weight (left and right) and maximal length. Absolute adrenal weight and length were analysed descriptively for each gland separately. In adult individuals, absolute adrenal measurements were summarised by sex and expressed as mean ± standard deviation (SD).

To account for inter-individual differences in body size, adrenal weight and length were additionally standardized by total body length and expressed as g/cm and cm/cm, respectively. Mean adrenal weight per individual was calculated as the average of left and right adrenal weights when both were available. Associations between mean adrenal weight and total body length were evaluated using Spearman’s rank correlation coefficient.

Histological morphometric analysis was performed on paraffin-embedded sections using calibrated measurement tools integrated into the Olympus BX51 microscope imaging system. Measurements included cortical and medullary thickness for each gland. A histological corticomedullary ratio (CM) was calculated for each gland using the following formula:

CM = (cortex_1_ + cortex_2_)/medulla

*Cortex_1_ and cortex_2_ represent the cortical thickness measured on both sides of the medulla.*

To evaluate potential lateral asymmetry, paired comparisons between left and right CM values were performed using the Wilcoxon signed-rank test. These values were then averaged per individual (mean of left and right glands when both were available, or single measurement if just one gland was measured), generating a single per-individual CM value for subsequent analyses. Averaged CM values were later compared between sexually immature and sexually mature animals to assess differences in corticomedullary organization using the Wilcoxon rank-sum test.

CM values were also compared according to cause-of-death category in sexually mature individuals to explore whether corticomedullary proportions differed between animals that died following a sudden external event and those that experienced more progressive pathological processes. Two categories were established: acute cause of death (Acute COD), including animals that died as a result of fishing interaction, and non-acute cause of death (Non-acute COD), comprising individuals in which death was attributed to infectious disease, parasitic disease, or medical/degenerative organ failure, representing more prolonged or insidious pathological conditions. The subset of sexually mature individuals included in these comparative analyses is detailed in Supplementary Table S2. Differences in CM between these groups were assessed using the Wilcoxon rank-sum test.

Normally distributed variables are presented as mean ± SD, whereas non-normally distributed variables are reported as median and interquartile range (IQR).

Statistical analyses were performed in R, and significance was set at *p* < 0.05.

### 2.5. Ultrastructural Study (Transmission Electron Microscopy)

For ultrastructural analysis, selected adrenal tissue samples from 4 individuals (one adult male, one adult female, one juvenile male and one juvenile female) measuring approximately 2 × 5 mm and less than 1 mm in thickness were obtained from the central transverse plane of the gland. Samples were rinsed in phosphate-buffered saline (PBS) and fixed overnight at 4 °C in 2.5% glutaraldehyde prepared in 0.1 M phosphate buffer (pH 7.4).

Post-fixation was performed in 1% osmium tetroxide in the same buffer, followed by dehydration through a graded ethanol series and embedding in Araldite resin. Semi-thin sections were stained with toluidine blue for orientation, and ultra-thin sections were contrasted with uranyl acetate and lead citrate.

Ultrastructural observations were carried out using a JEM 1400 transmission electron microscope (JEOL Ltd., Tokyo, Japan) at the Central Microscopy Research Facilities of the Universidad de Córdoba (Spain).

Morphometric analyses were performed using ImageJ, version 1.53, (National Institutes of Health, Bethesda, MD, USA). The parameters assessed included mean cell diameter of cortical and medullary cells, diameter of secretory granules in chromaffin cells, and mean diameter of lipid droplets and mitochondria in cortical cells.

For each individual and cellular compartment, quantitative measurements were obtained from well-preserved, non-overlapping cells in representative micrographs captured at comparable magnifications. For each parameter, a minimum of 3–5 measurements were performed across different cells and fields to avoid repetition bias. Mean values were calculated per individual. For the juvenile group (*n* = 2), measurements from both individuals were pooled to obtain group mean ± standard deviation (SD). For adult male and adult female categories (*n* = 1 each), values are presented as mean ± SD reflecting intra-individual variability only. All analyses were descriptive in nature.

## 3. Results

### 3.1. Gross Anatomy and Morphology

**Figure 1:**
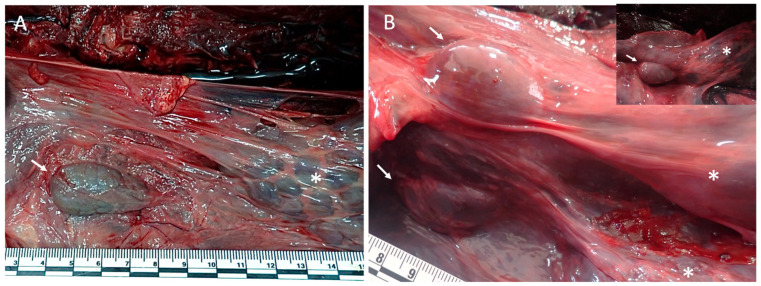
In situ anatomical position of the adrenal glands in *Delphinus delphis*. (**A**) Gross necropsy image showing the left adrenal gland (arrow) located craniomedial to the cranial pole of the left kidney (asterisk) within the abdominal cavity, partially embedded in perirenal connective tissue. (**B**) Gross necropsy image showing both adrenal glands (arrows) in situ following bilateral exposure, illustrating their symmetrical cranial relationship to the kidneys (asterisks) and overall topographical arrangement within the abdominal cavity. Inset: right adrenal gland (arrow) positioned craniomedial to the right kidney (asterisk) and located deeper within perirenal tissues; visualization requires partial reflection of the left kidney and surrounding connective tissue.

In all examined individuals, the adrenal glands were identified as paired organs located within the abdominal cavity. The glands were consistently found to be associated with the cranial pole of each kidney and were readily distinguishable from surrounding tissues by their compact appearance and well-defined capsule (Figure 1).

**Figure 2:**
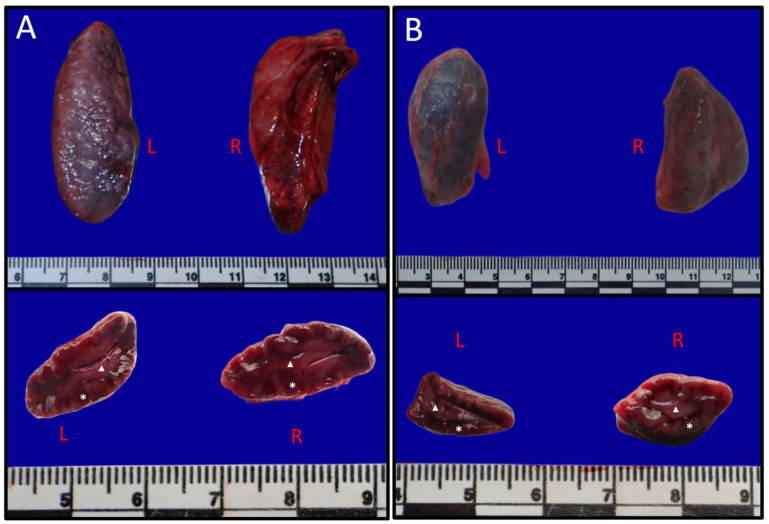
Gross morphology of the adrenal glands in sexually mature *Delphinus delphis*. (**A**) Adult male. (**B**) Adult female. Upper panels show left (L) and right (R) adrenal glands ex situ, demonstrating an elongated to oval morphology. Lower panels show mid-transverse sections of the corresponding glands, revealing clear cortico–medullary differentiation. The peripheral cortex (asterisk) appears darker, whereas the centrally located medulla (arrowhead) is lighter and more vascularized. Scale bars in cm.

Macroscopically, the adrenal glands were generally oval to elongated in shape, with a longitudinal axis of approximately two to three times their transverse width (Figure 2). The external surface was smooth and covered by a thin but distinct fibrous capsule. A mild asymmetry between left and right glands was observed in some individuals, primarily reflected in minor differences in size and contour, with the left adrenal tending to appear slenderer in many cases; however, overall gross organisation and cortico-medullary differentiation were comparable between sides.

On the mid-transverse section, two clearly demarcated regions were evident (Figure 2). The peripheral cortex formed the outer portion of the gland and appeared darker in colour, whereas the central medulla was lighter. The cortico-medullary boundary was grossly appreciable due to darker differences in colour and texture. Prominent vascular structures were visible within the medulla’s border on cut surface.

### 3.2. Histological Organization

**Figure 3:**
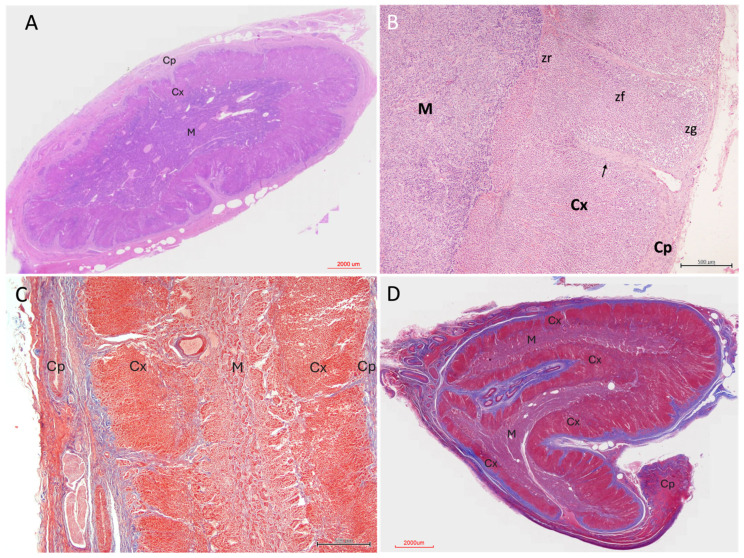
Architectural morphology of the adrenal gland in *Delphinus delphis*. (**A**) Submacroscopic view, hematoxylin–eosin (H&E), showing overall adrenal architecture with clearly distinguishable capsule, cortex, and medulla (M). (**B**) H&E (4×) showing capsule (Cp), cortex (Cx), and medulla (M). Connective tissue trabeculae (arrow) extend from the capsule into deeper regions. The three cortical zones are identifiable: zona glomerulosa (zg), zona fasciculata (zf), and zona reticularis (zr). (**C**) Masson’s trichrome (MT, 4×) demonstrating a collagen-rich capsule and connective tissue trabeculae penetrating the cortex, creating a pseudolobular appearance. Vessels and nerve fibers are visible within the connective tissue. (**D**) MT, submacroscopic view showing a non-typical cortico–medullary arrangement, with irregular distribution of cortical and medullary tissue and variable cortical compartmentalization.

Histological examination of the adrenal glands revealed a general organisation into an external capsule, a cortical component composed of three zones, and a central medulla (Figure 3).

Although most glands exhibited the typical peripheral cortical and central medullary organisation, some specimens displayed a more complex architectural arrangement. In these cases, cortex and medulla were distributed in irregular, alternating layers rather than forming a simple concentric pattern (Figure 3D), with focal intermingling of cortical and medullary components and the presence of morphologically distinct medullary regions within the same gland.

#### 3.2.1. Capsule and Stromal Organisation

**Figure 4:**
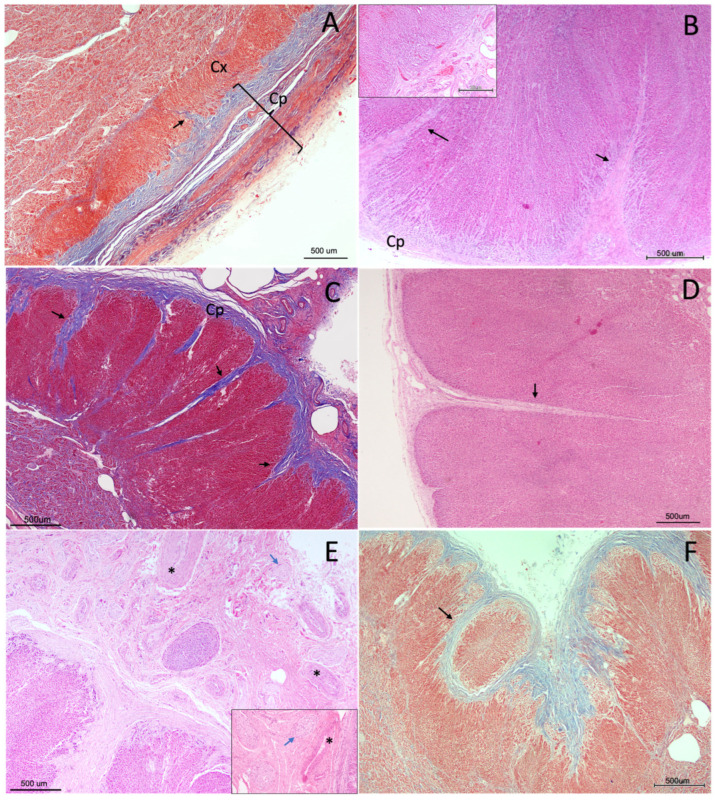
Capsular and stromal organisation of the adrenal gland in *Delphinus delphis*. (**A**) MT (4×) showing the fibrous capsule (Cp) and underlying cortex (Cx). Connective tissue trabeculae extend into the cortex, and thin septa are visible at the cortico–medullary junction, occasionally extending into the medulla (black arrow). (**B**) H&E (4×) showing trabeculae of variable thickness (black arrows) extending from the capsule (Cp) into the cortex. Inset: vascularization within capsular and subcapsular regions. (**C**) MT (2×) from a different individual showing a more developed trabecular framework (black arrows), including both shallow and deep trabeculae and thin septa extending into the medulla. (**D**) H&E (4×) showing a relatively thin capsule with a slender trabecula (black arrow) extending deeply into the zona fasciculata. (**E**) H&E (10×) detail of a thicker capsule containing small- and medium-caliber blood vessels (asterisks) and associated nerve fibres (blue arrows). Inset: higher magnification of neural elements. (**F**) MT (4×) showing focal capsular invagination partially surrounding cortical tissue (black arrow), forming a compartment that remains continuous with the surrounding cortex.

The adrenal gland was surrounded by a fibrous connective tissue capsule of variable thickness, which stained intensely with Masson’s trichrome, highlighting its collagenous composition (Figure 3B and Figure 4A,C). In some individuals, the capsule appeared thin and compact, whereas in others, it was thicker and more irregular.

The capsule was moderately vascularised and contained small- to medium-calibre blood vessels frequently accompanied by nerve fibres within the pericapsular connective tissue (Figure 3A and Figure 4B,E). These neurovascular structures extended inward along connective tissue trabeculae, forming a supporting stromal framework for the gland.

From the capsule, trabeculae projected irregularly into the cortex, forming a connective tissue network that supported and compartmentalised the adrenal parenchyma. These trabeculae were generally sparse and discontinuous, thinning as they progressed; however, in some specimens they penetrated deeply into the inner cortical zones, reaching the corticomedullary junction and occasionally extending into the medulla as thin septa (Figure 4). In these cases, trabecular extensions contributed to a pseudolobular organisation of the cortex, partially subdividing the parenchyma into irregular compartments.

Additionally, in some glands, small cortical regions adjacent to the capsule appeared partially or completely surrounded by capsular connective tissue. These areas remained morphologically continuous with the surrounding cortex and were interpreted as structural variations associated with capsular invagination or trabecular extension rather than discrete nodular formations. Within these regions, cortical zonation was reorganised concentrically around the connective tissue axis, with sequential re-establishment of the *zona glomerulosa*, *zona fasciculata*, and *zona reticularis* (Figure 4F).

#### 3.2.2. Cortical Architecture

The adrenal cortex was organised into three distinct zones, identifiable on the basis of cellular arrangement and staining characteristics: the *zona glomerulosa*, *zona fasciculata*, and *zona reticularis*.

Cortical vascularisation was evident as a network of thin-walled sinusoidal capillaries distributed between the cell cords. These vessels were most clearly appreciable in the *zona fasciculata*, where they ran longitudinally between radially arranged cellular columns (Figure 4B). The sinusoids were lined by flattened endothelial cells and frequently contained erythrocytes. Toward the cortico-medullary junction, vascular spaces became larger, more irregular, and more densely distributed, forming a transitional vascular zone between cortex and medulla. (Figure 3B). Blood vessels were commonly associated with connective tissue septa and trabeculae extending inward from the capsule, forming a supportive stromal framework within the cortex. Masson’s trichrome staining highlighted the collagenous components of these trabeculae and their close relationship with vascular structures (Figure 3C).

**Figure 5:**
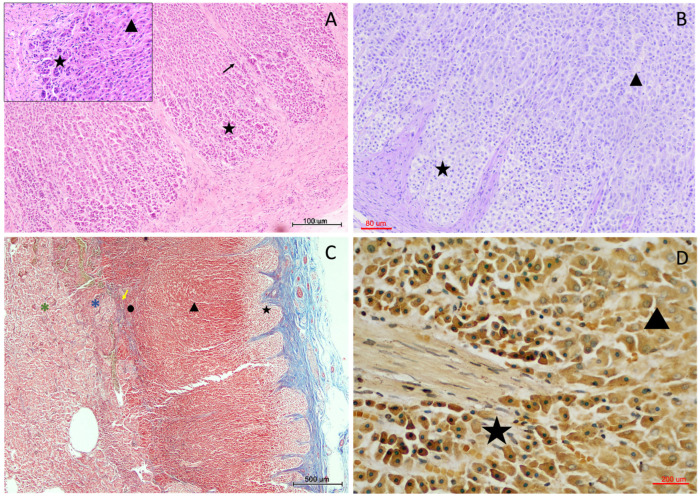
Histological characterisation of the zona glomerulosa in the adrenal cortex of *Delphinus delphis*. (**A**) H&E (10×) showing the zona glomerulosa (star) beneath the capsule, with trabeculae (black arrow) extending into the cortex. Inset: transition between zona glomerulosa (star) and zona fasciculata (triangle). (**B**) PAS-D (10×) showing zona glomerulosa (star) and zona fasciculata (triangle). (**C**) MT (4×) showing cortical zonation: zona glomerulosa (star), zona fasciculata (triangle), zona reticularis (circle), and cortico–medullary interface (yellow arrow). The medulla (asterisks) shows a dense vascular network and two chromaffin populations with differential staining (blue and green asterisks). (**D**) Grimelius (20×) showing zona glomerulosa (star) and adjacent zona fasciculata (triangle).

The outermost *zona glomerulosa* consisted of small, densely packed cells arranged in rounded or convex arched clusters beneath the capsule (Figure 5). These cells displayed relatively dark nuclei and scant cytoplasm in H-E staining. At higher magnification, cells exhibited a compact cytoplasmic profile with limited vacuolation and a relatively high nucleus-to-cytoplasm ratio compared with deeper cortical layers.

Periodic acid–Schiff–diastase (PAS-D) staining revealed limited cytoplasmic positivity in this zone. In contrast, Grimelius staining produced a slight argyrophilic reaction in the cell clusters of the *zona glomerulosa* (Figure 5D).

In the zona glomerulosa, smaller vascular profiles were observed interspersed among the rounded cellular clusters.

**Figure 6:**
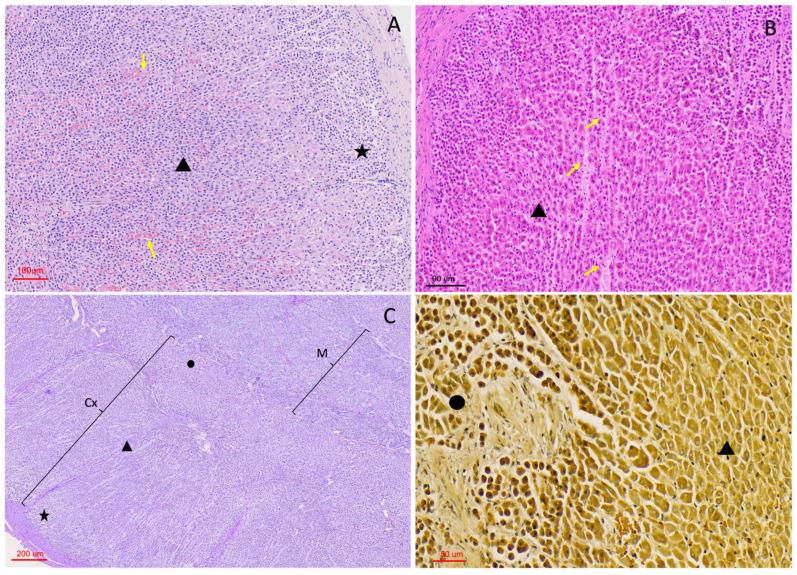
Histological organisation of the zona fasciculata and zona reticularis in the adrenal cortex of *Delphinus delphis*. (**A**) H&E (10×) showing zona fasciculata (triangle) beneath zona glomerulosa (star). (**B**) H&E (20×) detail of zona fasciculata cells (triangle) and associated sinusoidal spaces (yellow arrows). (**C**) PAS-D (4×) showing zona fasciculata (triangle), zona glomerulosa (star), zona reticularis (circle), and medulla (M). (**D**) Grimelius (20×) showing zona fasciculata (triangle) and zona reticularis (circle).

The *zona fasciculata* formed the largest portion of the cortex and was composed of large polygonal cells arranged in straight or slightly curved cords oriented radially toward the medulla (Figure 6). These cells exhibited abundant, lightly stained cytoplasm and occasionally clear vacuoles on H-E sections, consistent with high lipid content. Cells displayed a typical “spongiocyte” appearance, with cytoplasmic clearing due to lipid extraction during processing.

The innermost zona reticularis consisted of smaller, more irregularly arranged cells forming an interconnected network of short cellular cords. Compared with the zona fasciculata, these cells displayed more eosinophilic cytoplasm and darker nuclei. Cellular arrangement was less ordered, with cords forming a reticular or network-like pattern rather than parallel fascicles.

**Figure 7:**
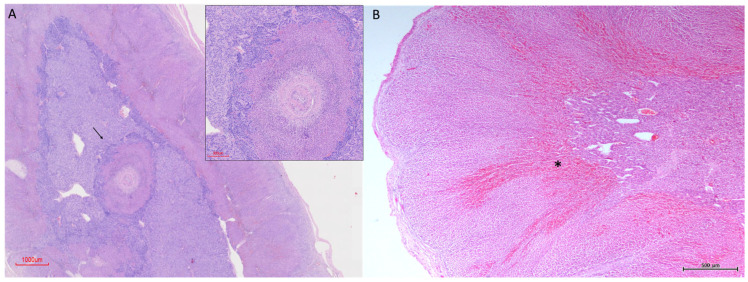
Cortical architectural reorganisation and corticomedullary vascularisation in the adrenal gland of *Delphinus delphis*. (**A**) H&E (3×) showing a large medullary vessel (arrow) surrounded by cortical tissue. Inset: detail of cortical organization around the vessel. (**B**) H&E (4×) showing vascular structures at the cortico–medullary junction (asterisk).

In one specimen, cortical tissue was observed surrounding medullary blood vessels, either as isolated cortical islands within the medulla or in association with deeply penetrating connective tissue trabeculae. In this area, local reorganisation of cortical architecture was evident, with cortical layering re-established around the trabecular or vascular axis while maintaining the sequential arrangement of the three cortical zones (Figure 7A).

In several specimens, marked vascular congestion was observed at the cortico-medullary junction. These changes were most evident in H-E stained sections and were localized primarily to the vascular transition zone between cortex and medulla. In some cases, focal erythrocyte extravasation within adjacent sinusoidal spaces was also observed (Figure 7B).

#### 3.2.3. Medullary Organisation

The adrenal medulla occupied the central region of the gland and was characterised by a dense vascular network, including numerous sinusoidal vessels and, in several specimens, one or more large-calibre venous channels consistent with a central medullary vein, that were not always located in a strictly central position (Figure 3A and Figure 7A). These vessels were readily visible on H-E and MT staining and exhibited a more defined vascular wall compared with surrounding sinusoids.

Chromaffin cells were arranged in cords or clusters surrounding vascular spaces. These clusters frequently adopted nest-like or rosette-like configurations, particularly in areas adjacent to vascular channels.

**Figure 8:**
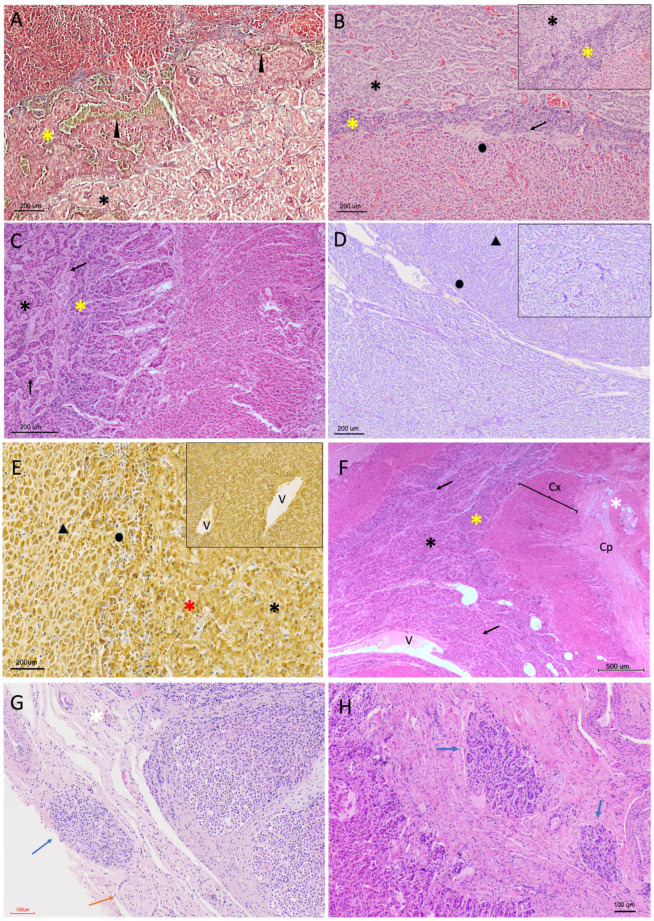
Medullary organisation and chromaffin cell distribution in the adrenal gland of *Delphinus delphis*. (**A**) MT (20×) showing peripheral (yellow asterisk) and central (black asterisk) chromaffin populations and medullary vessels (arrowheads). (**B**) H&E (10×) showing cortico–medullary interface with peripheral (yellow asterisk) and central (black asterisk) regions; adjacent cortex (circle) and connective interface (arrow) are visible. Inset: variation in peripheral medullary band thickness. (**C**) H&E (10×) showing medullary organization with the two different chromaffin populations (yellow and black asterisks) with connective elements (arrows). (**D**) PAS-D (10×) showing cortico–medullary transition with zona fasciculata (triangle), zona reticularis (circle), and medullary clusters. (**E**) Grimelius (10×) showing chromaffin populations (red and black asterisks) and adjacent reticular (circle) and fascicular (triangle) layers of the cortex. Inset: chromaffin clusters associated with vessels (V). (**F**) H&E (10×) showing medullary vessels (V), connective septa (arrows), the two different chromaffin populations (yellow and black asterisks), and adjacent cortex (Cx) and capsule (Cp). (**G**) H&E (10×; inset 20×) showing chromaffin-like cells within capsular connective tissue (blue arrow) associated with vascular (white asterisk) and neural elements (orange arrow). (**H**) H&E (14×) showing chromaffin clusters near the cortical boundary (blue arrows).

Two chromaffin cell populations could be distinguished based on cytoplasmic appearance and staining intensity. A peripheral population of chromaffin cells forming a “medullary band” at the cortico-medullary interface exhibited more basophilic cytoplasm, whereas more centrally located chromaffin cells appeared comparatively paler on H-E-stained sections (Figure 8).

Additionally, in a small number of cases, small aggregates of chromaffin-like cells were identified within the connective tissue of the capsule or along deep cortical trabeculae (Figure 8G,H). These cell clusters were sparse, focal, and morphologically similar to medullary chromaffin cells, appearing either adjacent to trabecular connective tissue or partially enclosed within capsular stroma, remaining structurally continuous with adjacent adrenal parenchyma.

### 3.3. Adrenal Morphometry

#### 3.3.1. Adrenal Weight and Length in Sexually Mature Individuals

Absolute adrenal measurements in sexually mature *Delphinus delphis* showed overlapping distributions between sexes. Mean left adrenal weight was 7.44 ± 2.85 g in females (*n* = 12) and 7.22 ± 2.36 g in males (*n* = 12). Mean right adrenal weight was 7.17 ± 2.14 g in females and 6.99 ± 2.70 g in males.

Absolute adrenal length followed a similar pattern. Mean left adrenal length was 4.73 ± 0.77 cm in females (*n* = 18) and 4.99 ± 1.67 cm in males (*n* = 15), while mean right adrenal length was 4.77 ± 0.82 cm in females and 5.29 ± 1.05 cm in males.

Absolute adrenal morphometry by sex is provided in Supplementary Figure S1.

#### 3.3.2. Body Size-Standardised Adrenal Morphometry Across Developmental Stages

**Figure 9:**
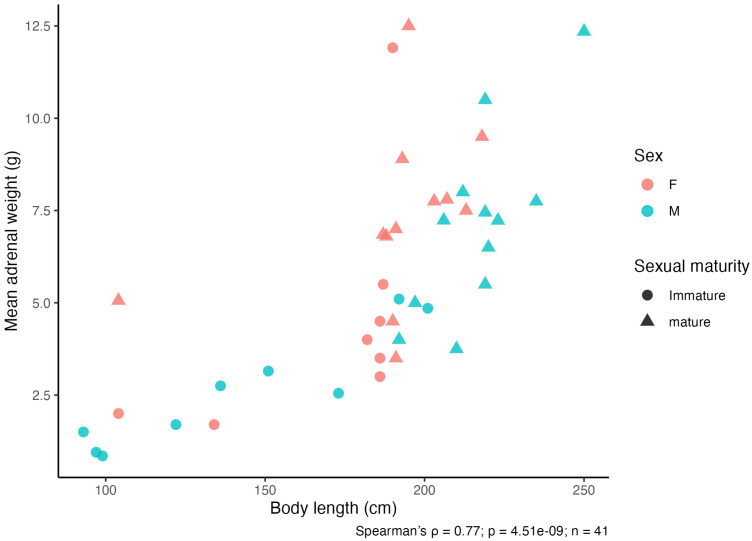
Relationship between body length and adrenal weight in *Delphinus delphis*. Scatterplot showing the association between total body length and mean adrenal weight (calculated as the average of left and right adrenal glands) across all individuals. The relationship was assessed using Spearman’s rank correlation (ρ = 0.77, *p* = 4.51 × 10^−9^).

The relationship between total body length and mean adrenal weight was assessed in individuals with complete body length data and adrenal weight measurement data (*n* = 41). Mean adrenal weight (calculated as the average of left and right glands when both were available) showed a positive association with body *length (Spearman’s ρ = 0.77, p = 4.51 × 10^−9^;* Figure 9*).*

When expressed relative to body length, adrenal weight and length were calculated for all individuals with available measurements. Sample sizes differed slightly between left and right glands due to incomplete data (Table 1).

#### 3.3.3. Corticomedullary Ratio (CM)

The histological corticomedullary ratio (CM) was calculated separately for left and right adrenal glands as the sum of cortical thickness measured on both sides of the medulla divided by medullary thickness. CM values were obtained for 50 left and 48 right adrenal glands (Table 2).

Paired comparison between left and right glands confirmed no significant difference (Wilcoxon signed-rank test: V = 294.5, *p* = 0.12), indicating the absence of lateral asymmetry.

In the absence of significant side-related differences, CM values were averaged per individual (mean of left and right glands when available), generating a single per-individual CM value for subsequent analyses.

**Figure 10:**
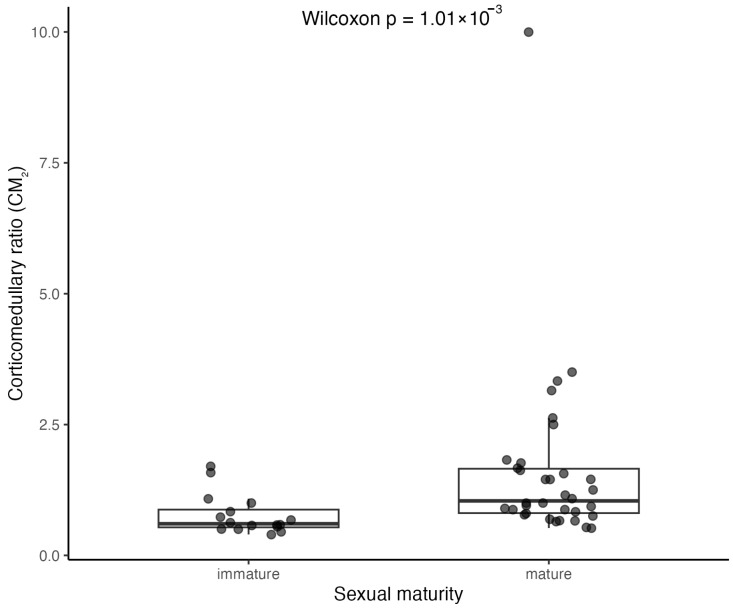
Corticomedullary ratio (CM) in relation to sexual maturity in *Delphinus delphis*. Boxplots showing per-individual CM values (mean of left and right adrenal glands when available) in sexually immature (*n* = 16) and mature (*n* = 34) individuals. Boxes represent the median and interquartile range (IQR), with individual data points overlaid.

When analysed according to sexual maturity, CM differed significantly between sexually immature (*n* = 16) and mature (*n* = 34) individuals (Wilcoxon rank-sum test: W = 113.5, *p* = 0.0010). Mature individuals exhibited higher median CM values (1.04, IQR 0.85) compared to immature individuals (0.60, IQR 0.34) (Figure S2 and Figure 10). 

**Figure 11:**
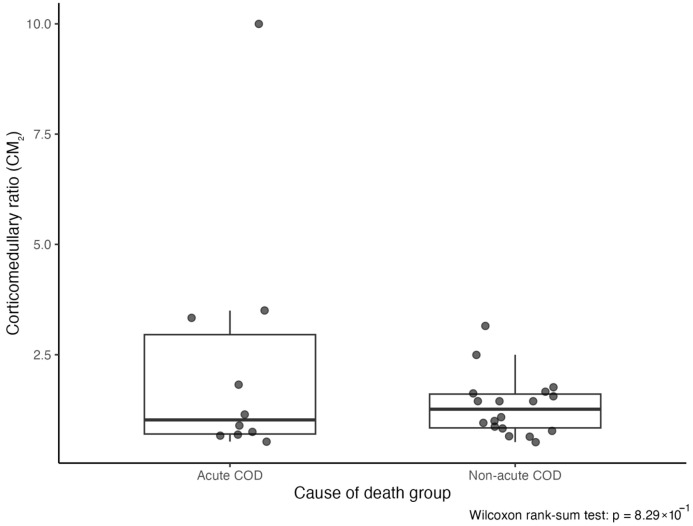
Corticomedullary ratio (CM) in adult *Delphinus delphis* according to cause of death. Per-individual CM values (mean of left and right adrenal glands) in sexually mature individuals classified as acute cause of death (COD; fishing interaction, *n* = 10) and non-acute COD (infectious, parasitic, or medical/degenerative causes; *n* = 18). Boxes represent the median and IQR, with individual data points shown. No significant difference was detected between groups (Wilcoxon rank-sum test: W = 95, *p* = 0.8291).

In sexually mature individuals, CM values were further evaluated in relation to cause of death (COD). Animals were classified as Acute COD (fishing interaction; *n* = 10) or Non-acute COD (infectious, parasitic, or medical/degenerative causes; *n* = 18). Median CM_2_ was 1.02 (IQR 2.25) in the Acute COD group and 1.27 (IQR 0.77) in the Non-acute COD group. The Acute COD group showed greater variability, largely influenced by one individual exhibiting an unusually high CM value. Despite a numerically higher median in the Non-acute COD group, no statistically significant difference was detected between groups (Wilcoxon rank-sum test: W = 95, *p* = 0.8291) (Figure S3 and Figure 11). 

### 3.4. Ultrastructural Study

**Figure 12:**
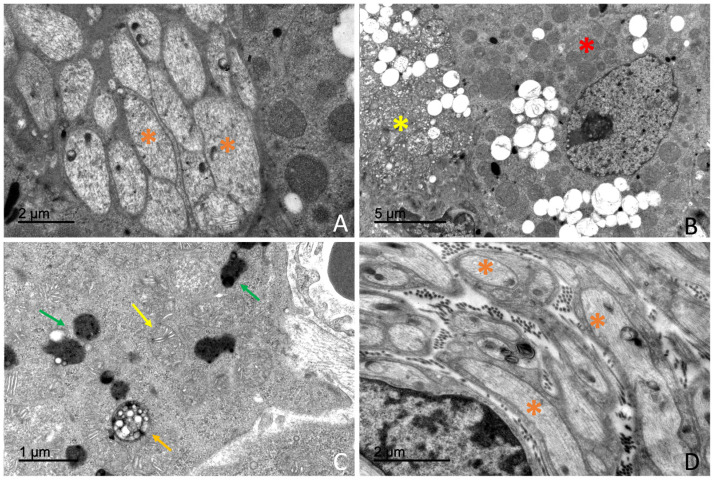
Transmission electron microscopy illustrating general ultrastructural features of the adrenal gland and associated innervation. (**A**) Amyelinated nerve fibers located within the interstitial space (orange asterisk). (**B**) Cortical spongiocyte showing cytoplasmic degeneration (yellow asterisk) adjacent to a morphologically normal cell (red asterisk). (**C**) Zona reticularis cell displaying an autophagosome (orange arrow), digitiform mitochondria (yellow arrow), and electron-dense lysosomes (green arrows). (**D**) Amyelinated nerve fibers in close association with adrenal parenchymal cells (orange asterisk).

Transmission electron microscopy (TEM) revealed a well-organized adrenal gland displaying a clear cortical–medullary differentiation. Distinct ultrastructural features were observed in each cortical zone as well as in the adrenal medulla, where two populations of chromaffin cells were identified based on the morphology of their secretory granules. A rich vascular network composed of fenestrated capillaries within the cortex and sinusoidal vessels within the medulla was observed. Endocrine cells were closely opposed to vascular endothelium. Amyelinated nerve fibres were observed in the interstitial spaces of the adrenal gland, in close association with both cortical and medullary cells (Figure 12).

#### 3.4.1. Adrenal Cortex

Transmission electron microscopy revealed that the adrenal cortex was organized into three distinct cellular zones: *the zona glomerulosa*, *zona fasciculata*, and *zona reticularis*. Each cortical zone was composed of cells with characteristic ultrastructural features, including differences in cytoplasmic organization, organelle abundance, and the presence of lipid droplets.

Ultrastructural morphometric measurements were performed to quantify mean cell diameter in each cortical zone and in medullary chromaffin cell types. Mean cell diameter (µm) values are summarised in Table 3.

##### *Zona* *Glomerulosa*

**Figure 13:**
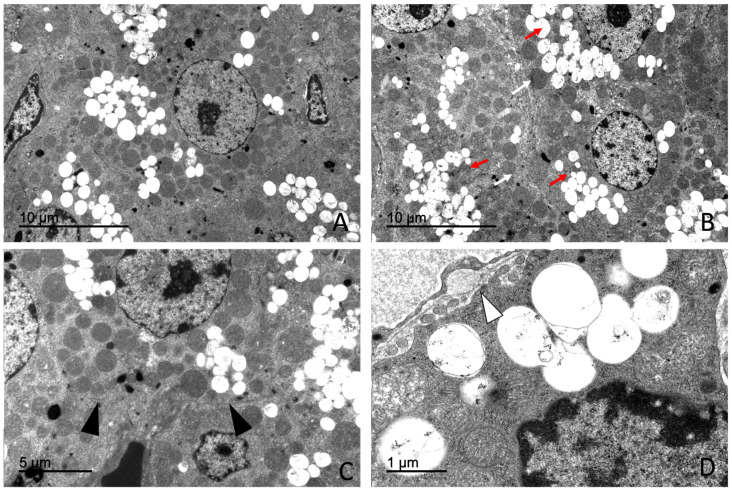
Transmission electron microscopy of the zona glomerulosa of the adrenal cortex in *Delphinus delphis*. (**A**) Glomerulosa cells arranged in compact, mosaic-like clusters. (**B**) Detail showing digitiform mitochondria (white arrows) and lipid droplets (red arrows). (**C**) Additional view of the characteristic mosaic organization (black arrowheads). (**D**) Glomerulosa cell in close association with a fenestrated capillary (white arrowhead).

Cells of the *zona glomerulosa* were arranged in compact clusters or glomeruli. Cells displayed a polygonal morphology and contained centrally located, round to oval nuclei with predominantly euchromatic chromatin and small heterochromatin aggregates (Figure 13).

The cytoplasm was characterized by abundant smooth endoplasmic reticulum, numerous lipid droplets, and a high density of mitochondria with tubular or digitiform cristae lacking elementary particles. Mitochondria exhibited a mean diameter ranging between 545 and 555 nm across age and sex categories. Lipid droplets displayed a mean diameter of approximately 645–660 nm, depending on the animal subgroup, and were observed either isolated or forming small clusters within the cytoplasm (Table S3).

##### *Zona* *Fasciculata*

**Figure 14:**
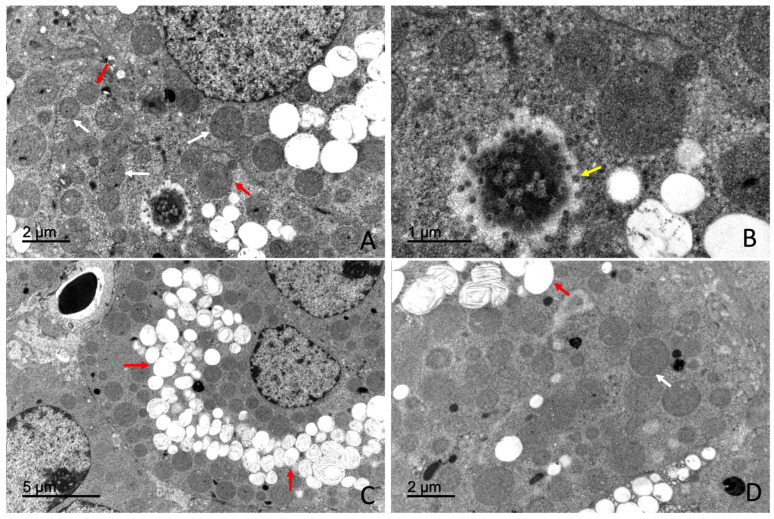
Transmission electron microscopy showing characteristic features of the zona fasciculata in *Delphinus delphis*. (**A**) Fasciculata cell with abundant mitochondria (white arrows) and lipid droplets (red arrows). (**B**) Nucleus exhibiting prominent nuclear pores (yellow arrow). (**C**) Spongiocyte with numerous lipid droplets occupying most of the cytoplasm (red arrows). (**D**) Spongiocyte displaying abundant mitochondria (white arrow) and lipid droplets of variable size (red arrows).

Cells of the zona fasciculata were large and arranged in cords. These cells exhibited a markedly electron-lucent cytoplasm (Figure 14).

Numerous lipid droplets occupied extensive areas of the cytoplasm. These lipid droplets were membrane-bound, showed an electron-lucent content and displayed a mean diameter of approximately 660–680 nm, depending on the animal subgroup. The cytoplasm was characterized by the presence of an extensive smooth endoplasmic reticulum occupying large cytoplasmic regions. Numerous mitochondria with digitiform cristae lacking elementary particles showing an average diameter of approximately 575–580 nm were also observed (Table S3).

Rough endoplasmic reticulum and Golgi complexes were present, whereas lysosomes were scarce. Nuclei were generally round to oval and predominantly euchromatic.

Occasional fasciculata cells exhibited ultrastructural features consistent with cytoplasmic degeneration, including vacuolization and organelle disorganization (Figure 12B).

##### *Zona* *Reticularis*

**Figure 15:**
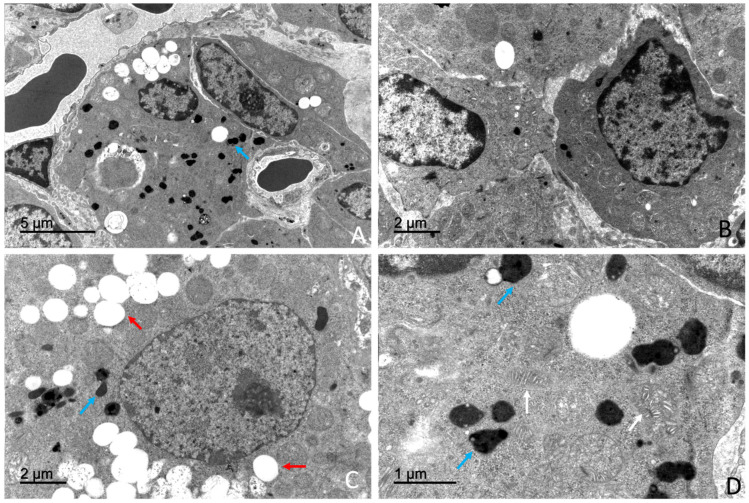
Transmission electron microscopy of zona reticularis cells in *Delphinus delphis*. (**A**) Reticular cells with abundant electron-dense lysosomes (blue arrow). (**B**) Irregular arrangement of zona reticularis cells within the cortex. (**C**) Cytoplasm containing clusters of lipid droplets (red arrows) and lysosomes (blue arrow). (**D**) Reticular cell showing digitiform mitochondria (white arrows) and dense lysosomes (blue arrow).

Cells of the zona reticularis were smaller and more compactly arranged than those of the zona fasciculata. The cytoplasm exhibited increased electron density (Figure 15). 

Compared with glomerulosa and fasciculata cells, zona reticularis cells showed a reduced abundance of smooth endoplasmic reticulum, lipid droplets, and mitochondria, although mitochondria displayed a pleomorphic morphology. Rough endoplasmic reticulum and Golgi complexes were present. The presence of autophagosomes was observed occasionally (Figure 14C).

A high number of pleomorphic lysosomes was observed and represented a distinctive morphological feature of these cells (Figure 15). Electron-dense inclusions of variable size and morphology, consistent with residual bodies or lipofuscin-like material, were frequently observed in the cytoplasm. Nuclei showed irregular contours and an increased proportion of heterochromatin.

Morphometric analysis showed that lipid droplets in zona reticularis cells exhibited mean diameters of approximately 650–670 nm, while mitochondria presented mean diameters of approximately 574–584 nm (Table S3).

**Figure 16:**
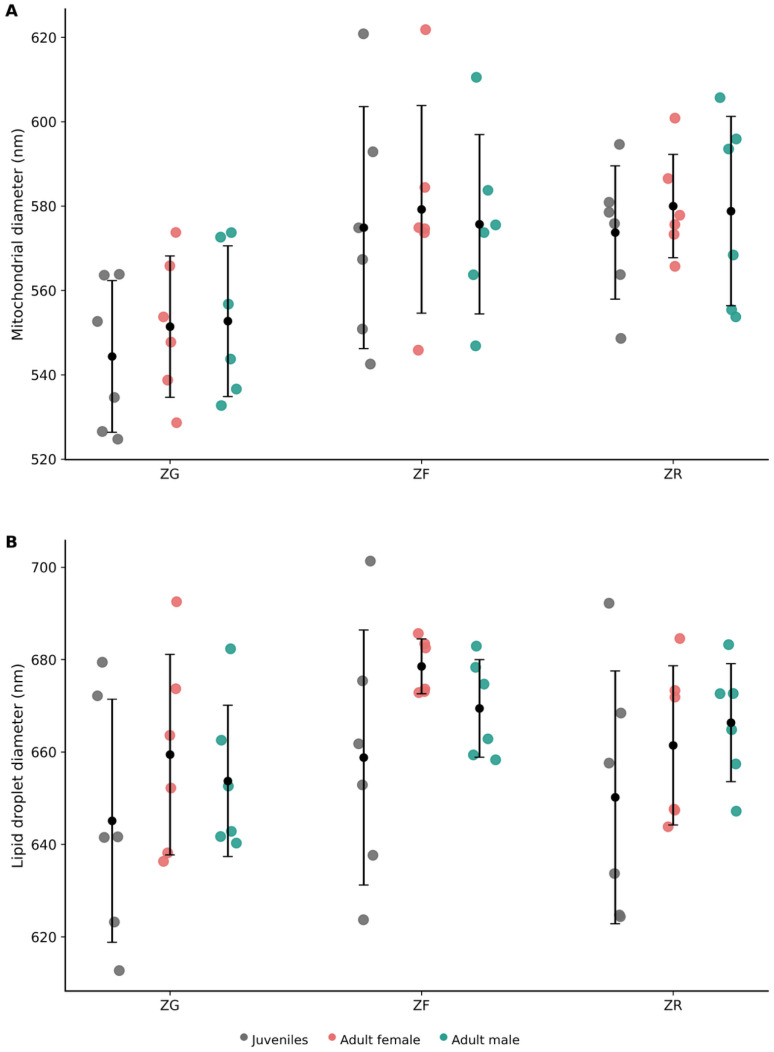
Ultrastructural morphometric analysis of adrenal cortical organelles in *Delphinus delphis*. (**A**) Mean mitochondrial diameter (nm) in zona glomerulosa (ZG), zona fasciculata (ZF), and zona reticularis (ZR). (**B**) Mean lipid droplet diameter (nm) in the same cortical zones. Each dot represents an individual measurement (*n* = 6 per group). Black symbols indicate mean ± SD. Juvenile values represent pooled measurements (*n* = 2), whereas adult female and male values correspond to single individuals (*n* = 1 each) and reflect intra-individual variability.

A comparative overview of mitochondrial and lipid droplet diameters across cortical zones is presented in Figure 16. 

#### 3.4.2. Adrenal Medulla

Transmission electron microscopy revealed that the adrenal medulla was composed predominantly of chromaffin cells arranged in close association with vascular structures. Two chromaffin cell populations were identified based on their ultrastructural characteristics, particularly the morphology and electron density of their secretory granules.

Morphometric analyses were performed to quantify granule size and mean cell diameter across animal subgroups. Mean cell diameter values for medullary chromaffin cells are presented in Table S4.

##### *Chromaffin* *Cells Type A*

**Figure 17:**
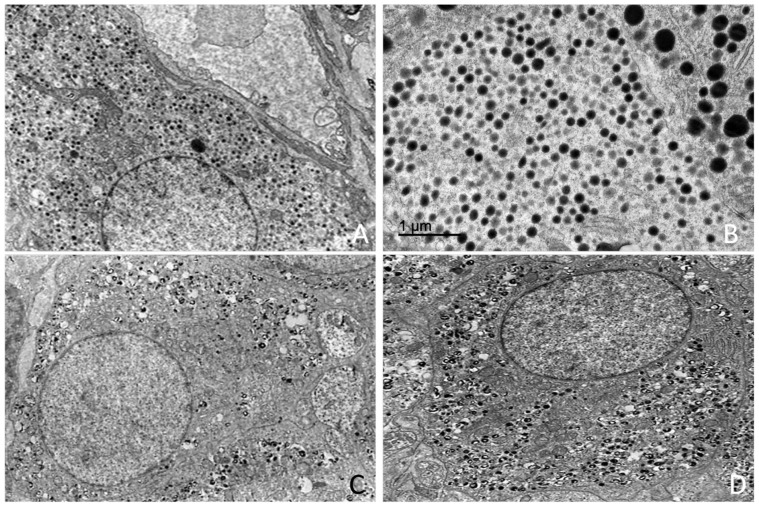
Transmission electron microscopy of chromaffin cells in the adrenal medulla in *Delphinus delphis*. (**A**,**B**) Type A (adrenaline) chromaffin cells characterized by numerous small, homogeneous, electron-dense secretory granules. (**C**,**D**) Type NA (noradrenaline) chromaffin cells showing pleomorphic granules with heterogeneous electron density and a prominent electron-lucent halo.

Type adrenaline (A) chromaffin cells were characterized by a cytoplasm densely filled with numerous membrane-bound secretory granules (Figure 17A,B). These cells were organized in a palisade arrangement below the cortical area.

The secretory granules were predominantly electron-dense, relatively homogeneous in size, and exhibited a narrow or poorly defined electron-lucent halo. Granules were diffusely distributed throughout the cytoplasm. Morphometric analysis revealed granule diameters of 375.05 ± 29.83 nm in juveniles, 383.52 ± 24.85 nm in the adult female, and 385.56 ± 17.62 nm in the adult male (Table S4).

The cytoplasm also contained rough endoplasmic reticulum and Golgi complexes. Mitochondria were present and did not display distinctive ultrastructural features. Nuclei were generally large and predominantly euchromatic, with finely dispersed chromatin.

##### *Chromaffin Cells* *Type NA*

Type noradrenaline (NA) chromaffin cells had a scattered distribution and exhibited a cytoplasm densely packed with membrane-bound secretory granules showing marked heterogeneity in size and electron density (Figure 17C,D).

Secretory granule diameter was slightly smaller in type NA cells compared to type A cells across all animal subgroups. These were characterized by an eccentrically located electron-dense core surrounded by a wide and well-defined electron-lucent halo. Granules were abundantly distributed throughout the cytoplasm and occasionally formed clusters. Granule diameter measured 364.14 ± 32.65 nm in juveniles, 362.50 ± 27.14 nm in the adult female, and 363.71 ± 31.59 nm in the adult male (Table S4).

The cytoplasm contained rough endoplasmic reticulum and Golgi complexes, mainly in perinuclear regions. Mitochondria were present without distinctive ultrastructural features. Nuclei were large, round to oval, and predominantly euchromatic.

**Figure 18:**
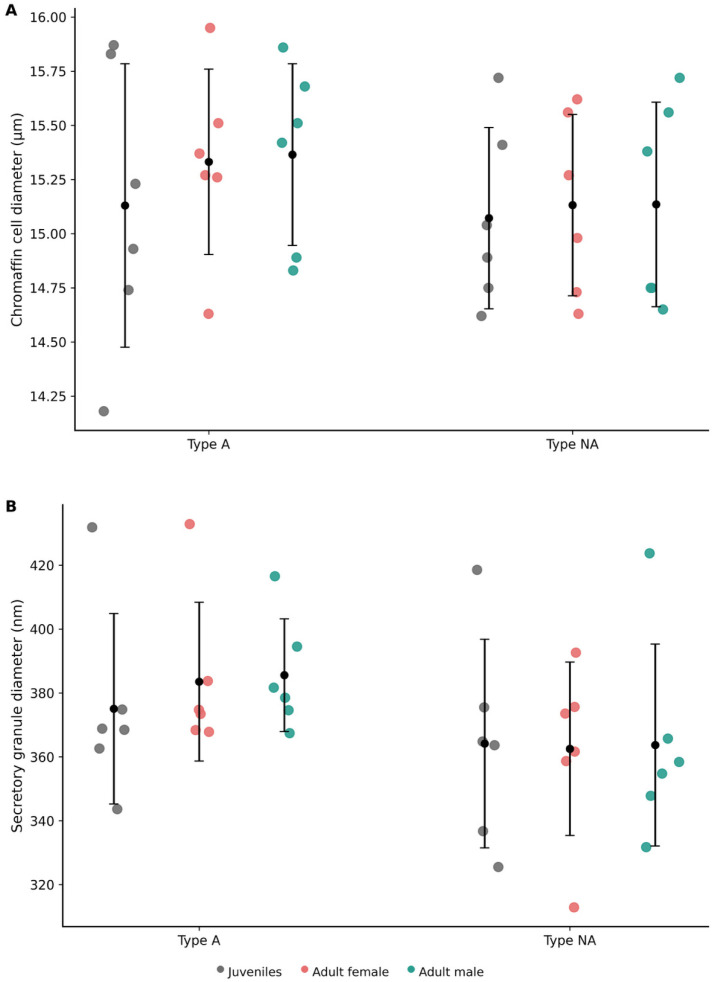
Ultrastructural morphometric analysis of adrenal medullary chromaffin cells in *Delphinus delphis*. (**A**) Mean chromaffin cell diameter (µm) in type A (adrenaline) and type NA (noradrenaline) cells. (**B**) Mean secretory granule diameter (nm) in both chromaffin cell types. Each dot represents an individual measurement (*n* = 6 per group). Black symbols indicate mean ± SD. Juvenile values represent pooled measurements (*n* = 2), whereas adult female and male values correspond to single individuals (*n* = 1 each) and reflect intra-individual variability.

Mean chromaffin cell diameter values were comparable across age and sex categories in both cell types. Granule diameter was consistently higher in type A cells than in type NA cells across all subgroups. A comparative morphometric overview is presented in Figure 18.

## 4. Discussion

This study integrates gross, histological, morphometric, and ultrastructural analyses to examine adrenal gland organization in the short beaked common dolphin (*Delphinus delphis*). The combined approach enables intra-specific evaluation and comparative interpretation across delphinids.

### 4.1. Gross and Histological Architecture of the Gland

The adrenal glands of *Delphinus delphis* conformed to the general odontocete pattern Connective tissue septation followed the typical delphinid arrangement: capsular trabeculae extended toward the cortico-medullary boundary and occasionally continued as fine septa into the medulla, similar to descriptions in other dolphin species [24,25,26,28,37,38]. Cross-sections were predominantly oval, and left and right glands were broadly symmetrical, with only minor inter-individual variation. This contrasts with the more polygonal outline described in the common bottlenose dolphin (*Tursiops truncatus*) [25]. This pattern supports their interpretation as architectural variants rather than structural abnormalities.

The presence of cortical projections extending into the medulla, often associated with penetrating vessels, has also been described in other mammals as well as delphinids, where penetrating vessels ensheathed by cortical layers extend into the medulla [25,39]. Such vascular-associated cortical invaginations are generally interpreted as growth-related architectural variants [24]. Although somatic growth stabilizes after sexual maturity in cetaceans, endocrine organs may continue to increase in mass at a slower rate [24,40]. The presence of this configuration in a large, sexually mature individual therefore supports a developmental rather than pathological interpretation.

From a developmental perspective, the adrenal gland reflects the distinct embryological origins of cortex and medulla [41]. Irregular cortical projections, intramedullary cortical nodules, and vascular-associated cortical inclusions have been described in humans, rodents, and domestic ungulates, where they are typically considered non-pathological variants linked to developmental processes or postnatal structural development [42,43].

Occasional clusters of chromaffin-like cells were identified within the capsule and along cortical trabeculae. Similar capsular aggregates have been described in the bottlenose dolphin and interpreted as accessory adrenal tissue [25,26]. In terrestrial mammals, extra-medullary chromaffin tissue has been reported along vascular pathways and within connective septa, often linked to developmental migration [44,45,46]. Given the close cortico–medullary interaction within the adrenal gland, where cortical glucocorticoids influence catecholamine synthesis through paracrine mechanisms [47,48], the proximity of chromaffin cells to cortical tissue and penetrating vessels in *Delphinus delphis* likely reflects functional integration rather than structural irregularity.

Cortical zonation followed the classical three-layered organization. In contrast to the findings in common bottlenose dolphins, where a thin zona intermedia was described beneath the zona glomerulosa, no distinct intermediate layer was identified here. Transitional cortical layers have been reported inconsistently in other mammals, including humans [49,50,51], and their absence in *Delphinus delphis* supports interspecific variability in adrenal cortical microarchitecture within Delphinidae.

An unexpected finding was the presence of argyrophilic staining in the zona glomerulosa using the Grimelius method. This stain is typically associated with neuroendocrine cells of the adrenal medulla, and its expression in the cortex is not commonly reported. In the present study, the reaction in the zona glomerulosa was less consistent and less intense than in the medulla, suggesting a different biological or technical basis. Although argyrophilia is often linked to neuroendocrine differentiation, it is not entirely specific and may reflect cytoplasmic components capable of binding silver salts. Previous studies have reported neuroendocrine-related features in the adrenal cortex under certain conditions, but their functional significance remains unclear [52,53]. Therefore, in the absence of immunohistochemical confirmation, this finding should be interpreted with caution.

A peripheral band of intensely basophilic chromaffin cells was consistently observed at the cortico-medullary interface, corresponding to the “medullary band” described in several delphinid species, including common bottlenose dolphins (*Tursiops truncatus*), pantropical spotted dolphin (*Stenella attenuata*) and in the spinner dolphin (*Stenella longirostris*) [24,25,26]. This organization supports the presence of distinct chromaffin cell populations and suggests that this arrangement represents a conserved pattern rather than a cetacean-specific specialization [54,55,56]. However, in the absence of immunohistochemical confirmation, the functional identity of these two chromaffin cell populations could not be definitively established at the morphological level.

In several individuals, vascular congestion was observed at the cortico-medullary junction, occasionally accompanied by focal erythrocyte extravasation. These changes were not associated with disruption of adrenal architecture and were localized primarily to the vascular transition zone between cortex and medulla. The adrenal gland exhibits a centripetal vascular organization in which cortical sinusoidal blood flows toward the medulla before draining through the central vein, creating a vascular convergence zone at the cortico-medullary interface [57]. During acute physiological stress, simultaneous activation of the HPA axis and the sympathoadrenal system is accompanied by increased adrenal perfusion and intense catecholamine release, processes that may promote sinusoidal dilation and vascular congestion [58,59]. Comparable adrenal vascular alterations have been described in other wildlife species exposed to extreme physiological stress, including capture or stranding events, where they are interpreted as part of the systemic stress response [60]. Further integrative investigations combining endocrine, vascular, and histopathological parameters would be required to clarify the biological significance of these findings in stranded cetaceans.

The present dataset comprised a substantial intra-specific sample collected over an extended period and including individuals that died under both acute and chronic conditions. This allowed for an evaluation of the adrenal architecture across diverse physiological and pathological contexts within a single species. Previous studies suggested that architectural irregularities may be more frequent in stranded *T. truncatus* than in exclusively bycaught *Stenella* species [25,26]; however, those comparisons involved different species as well as different mortality contexts. Within the *Delphinus delphis* dataset examined here, no consistent increase in cortical or medullary irregularities was detected across mortality categories, and overall glandular organization remained stable.

Collectively, these observations indicate that adrenal morphology in *Delphinus delphis* is primarily shaped by species-specific structural patterns while retaining a degree of architectural flexibility.

### 4.2. Morphometry

Adrenal morphometry in *Delphinus delphis* demonstrated bilateral symmetry, with no consistent lateral differences in gland size or corticomedullary proportions. The corticomedullary ratio approximated unity in most individuals, indicating near-equivalent cortical and medullary thickness. This balanced organization contrasts with the more cortex-dominant patterns reported in other delphinids, including *Tursiops truncatus* and *Stenella* species [25,26], highlighting interspecific variability in adrenal proportional architecture within Delphinidae. Methodological differences may also contribute to variation among studies, as the present work employed standardized mid-transverse linear measurements, whereas others have used area-based or multivariate approaches [10,29].

Adrenal dimensions scaled proportionally to body length, supporting the interpretation that adrenal growth follows general somatic development, as previously reported in cetaceans [10,40,61]. Importantly, corticomedullary proportions differed significantly between immature and mature individuals, with mature dolphins exhibiting higher CM values overall. This pattern indicates ontogenetic reorganization of adrenal architecture during development. Such changes are consistent with the distinct developmental dynamics of the adrenal cortex and medulla: the cortex, of mesodermal origin, undergoes substantial postnatal remodeling and remains highly responsive to endocrine regulation, whereas the medulla, derived from neural crest cells, tends to exhibit a comparatively more stable structural organization. Accordingly, variation in corticomedullary ratio is likely driven primarily by cortical growth and differentiation rather than medullary expansion. In functional terms, this ontogenetic shift may reflect progressive maturation of the hypothalamic–pituitary–adrenal (HPA) axis, with structural differentiation of the cortex accompanying the establishment of glucocorticoid production capacity and adaptive stress responses [41,62]. In contrast, maturity-related differences in corticomedullary ratio have not been consistently reported in other delphinids, including *Tursiops truncatus* [25], although differences in sampling design, age classification, and morphometric methodology may partly account for this discrepancy.

No consistent differences in corticomedullary ratio were detected between adult individuals who died from acute events and those who died following more progressive pathological processes. Although minor numerical variation was observed, distributions largely overlapped, indicating comparable corticomedullary architecture across mortality categories in this species. This contrasts with earlier observations in other dolphin species, in which bycaught individuals and those affected by chronic disease were reported to exhibit distinct adrenal proportional patterns [10,29],

Together, these findings suggest that adrenal proportional architecture may be influenced by species-specific and multifactorial physiological processes that extend beyond simple cause-of-death categorization. In Harbor porpoises (*Phocoena phocoena*), beluga whales (*Delphinapterus leucas*) and common bottlenose dolphins (*Tursiops truncatus*), cortical hyperplasia and adrenal enlargement have been described in association with chronic disease, contaminant exposure, and environmental stressors [12,29,63,64]. Similar patterns have been reported in other wildlife species exposed to prolonged anthropogenic or ecological pressures, and experimental models demonstrate that sustained ACTH stimulation or chronic stress paradigms can induce adrenocortical hypertrophy [65,66]. However, adrenal responses are not uniformly hypertrophic; cortical thinning, reduced adrenal mass, or zona-specific atrophy have also been documented under certain conditions [67,68]. In some models, sustained stress altered the HPA-axis sensitivity without consistent macroscopic enlargement [69,70,71,72,73], highlighting the complexity and context-dependence of endocrine–morphological relationships.

Accordingly, interpreting corticomedullary ratio as a direct structural proxy for chronic stress requires caution. Developmental stage, age-related remodeling, reproductive status, metabolic condition, and species-specific regulatory mechanisms may substantially influence adrenal proportional organization [74,75] The significant differences observed between immature and mature individuals in the present study further emphasize the impact of developmental stage on corticomedullary organisation.

While classification of mortality as “acute” or “chronic” provides a useful epidemiological framework, it may not fully capture the complexity of an individual’s endocrine history. Animals that die from sudden events may have experienced prior physiological challenges, whereas those assigned to chronic pathological categories may exhibit heterogeneous or fluctuating endocrine states. Within this intra-specific dataset, where age class and sexual maturity were explicitly considered, corticomedullary proportions in adult *Delphinus delphis* did not vary consistently between mortality categories. These findings suggest that, in this species, corticomedullary ratio appears more strongly influenced by developmental processes than by terminal pathological context, and its interpretation should consider broader biological and physiological context.

The use of stranded individuals as study material inherently introduces potential sources of bias, including post-mortem autolysis and physiological alterations associated with agonal processes, terminal stress, or disease. Although only well-preserved specimens were selected to minimize these effects, subtle tissue changes cannot be entirely excluded. Autolysis may particularly affect cellular detail and staining characteristics, while terminal physiological conditions may influence adrenal morphology, especially in relation to cortical activity and medullary function.

However, the consistency of the structural organization observed across individuals, together with the preservation of key histological and ultrastructural features, supports the reliability of the findings presented here. Moreover, stranding networks represent one of the most valuable sources of biological material for the study of cetaceans, given the inherent limitations associated with studying free-ranging individuals [76,77,78]. In many cases, animals strand alive or die shortly before recovery, allowing for reduced post-mortem intervals and improved tissue preservation. Furthermore, the application of standardized decomposition codes provides an objective framework to assess tissue quality and minimize interpretative bias [36,79].

### 4.3. Ultrastructural Organization of the Adrenal Gland

Transmission electron microscopy confirmed that the adrenal gland of *Delphinus delphis* largely conforms to the conserved mammalian ultrastructural framework, particularly in steroidogenic cortical organization and chromaffin cell architecture. Cells of the zona fasciculata exhibited abundant smooth endoplasmic reticulum, numerous mitochondria with tubular cristae, and prominent lipid droplets, consistent with the classical “spongiocyte” morphology described across mammals and associated with glucocorticoid biosynthesis [80,81,82]. Zona reticularis cells displayed comparatively greater cytoplasmic electron density and increased lysosomal components, features consistent with androgen precursor synthesis and terminal differentiation described in other vertebrate models [83], indicating the preservation of core steroidogenic ultrastructural features within *Odontoceti*.

Within the adrenal medulla, two morphologically distinct chromaffin cell populations were identified based on secretory granule morphology and electron density. One population exhibited relatively homogeneous, electron-dense granules with narrow or poorly defined halos, whereas the second displayed pleomorphic granules with eccentrically located dense cores surrounded by a well-defined electron-lucent halo. This chromaffin granule heterogeneity corresponds to the classical ultrastructural distinction between epinephrine- and norepinephrine-producing chromaffin cells described in mammalian medullary studies [45,84,85]. Contemporary reviews continue to support granule morphology as a reliable ultrastructural correlate of catecholamine phenotype in the absence of immunohistochemical confirmation [55]. Although definitive functional assignment in *Delphinus delphis* would require direct immunolabeling, the granule characteristics observed here strongly support the presence of both catecholaminergic cell populations. Together, these observations confirm that adrenal ultrastructure in *Delphinus delphis* follows conserved mammalian organizational principles while providing novel species-specific ultrastructural insights.

## 5. Conclusions

This study provides the first integrated gross, histological, morphometric, and ultrastructural characterization of the adrenal gland in *Delphinus delphis*. Adrenal architecture in this species aligns with conserved mammalian organizational principles while also exhibiting species-specific structural patterns within Delphinidae.

Corticomedullary proportions were influenced by ontogenetic stage but did not vary consistently according to mortality category, highlighting the importance of developmental context in interpreting adrenal morphology. Ultrastructural findings confirmed the preservation of steroidogenic and catecholaminergic cellular organization, including distinct chromaffin cell populations.

Given the integrative and multifactorial regulation of the HPA axis, structural parameters should not be interpreted in isolation as direct indicators of stress or dysfunction. Instead, they must be considered within the broader context of species-specific organization, developmental dynamics, and physiological state. Continued investigation of adrenal morphology in conjunction with functional, endocrine, and comparative approaches will be essential to further elucidate the complex neuroendocrine regulation underlying stress responses in cetaceans.

By defining structural baselines for *Delphinus delphis*, this work establishes a robust reference framework for future comparative studies and for the evaluation of endocrine-related findings in health assessment and stranding investigations, supporting a biologically contextualized interpretation of adrenal morphology rather than reducing structural variation to a single proxy of stress.

## Figures and Tables

**Table 1:** Body size–standardised adrenal morphometry in *Delphinus delphis*. Adrenal weight (g/cm) and length (cm/cm) expressed relative to total body length across all individuals. Values are presented as mean ± standard deviation (SD). Measurements were standardized by body length to account for inter-individual variation. Sample sizes (n) are reported separately for left and right glands due to incomplete measurements in some individuals.

Body Size—Standardised Adrenal Morphometry in Delphinus Delphis					
Adrenal weight and length expressed relative to body length					
n (Left)	n (Right)	Adrenal weight—Left (g/cm)—mean ± SD	Adrenal weight—Right (g/cm)—mean ± SD	Adrenal length—Left (cm/cm)—mean ± SD	Adrenal length—Right (cm/cm)—mean ± SD
41	40	0.0295 ± 0.0141	0.0299 ± 0.0133	0.0236 ± 0.0067	0.0248 ± 0.0062

Values are expressed as mean ± standard deviation.

**Table 2:** Left and right corticomedullary ratio (CM) descriptive statistics in *Delphinus delphis*. Descriptive statistics of the histological corticomedullary ratio (CM), presented separately for left and right adrenal glands. Values are expressed as median and interquartile range (IQR). Sample sizes (n) are reported independently for each side due to incomplete measurements. These data support the assessment of lateral symmetry prior to side-averaged analyses.

Corticomedullary Ratio (CM)					
n (Left)	Median (Left)	IQR (Left)	n (Right)	Median (Right)	IQR (Right)
50.00	0.94	0.81	48.00	1.00	0.83

**Table 3:** Mean cell diameter (µm) of adrenal cortical and medullary cells in *Delphinus delphis*. Values are expressed as mean ± standard deviation (SD). Juvenile values represent pooled measurements from two individuals (*n* = 2), whereas adult female and adult male values correspond to single individuals (*n* = 1 each) and reflect intra-individual variability.

Cell Type	Juveniles	Adult Female	Adult Males
Zona glomerulosa	16.34 ± 0.46	16.61 ± 0.98	16.48 ± 0.62
Zona fasciculata	16.66 ± 0.79	16.75 ± 0.51	16.81 ± 0.78
Zona reticularis	16.06 ± 0.36	16.20 ± 0.62	16.16 ± 0.51

## Data Availability

The original contributions presented in this study are included in the article/Supplementary Material. Further inquiries can be directed to the corresponding author(s).

## Supplementary Materials

The following supporting information can be downloaded at: https://www.mdpi.com/article/10.3390/vetsci13040348/s1, Table S1. Complete morphometric and histomorphometric dataset of *Delphinus delphis* included in this study; Table S2. Sexually mature *Delphinus delphis* included in cause-of-death comparative analyses; Figure S1. Absolute adrenal morphometry in sexually mature *Delphinus delphis* by sex; Figure S2. Comparison of corticomedullary ratio (CM) according to sexual maturity in *Delphinus delphis*; Figure S3. Comparison of corticomedullary ratio (CM) between acute and non-acute cause-of-death (COD) groups in sexually mature *Delphinus delphis*; Table S3. Mean diameter (nm) of mitochondria and lipid droplets in the adrenal cortex of *Delphinus delphis*; Table S4. Mean cell diameter (µm) of adrenal medullary cells in *Delphinus delphis*; Supplementary Document S1. Administrative authorization For the handling of stranded specimens on the Canary Islands coast, the collection and custody of their biological samples, and the performance of necropsies for scientific and conservation purposes.

## Abbreviations

The following abbreviations are used in this manuscript:

HPA	Hypothalamic-pituitary-adrenal
CM	Corticomedullary
COD	Cause of death
TEM	Transmission electron microscopy
IUSA	Instituto Universitario de Sanidad Animal y Seguridad Alimentaria
ULPGC	Universidad de Las Palmas de Gran Canaria
HE	Hematoxylin-eosin
MT	Massons’s trichrome
PAS	Periodic acid–Schiff
PAS-D	Periodic acid–Schiff-Diastase
IQR	Interquartile range
SD	Standard deviation
PBS	Phosphate-buffered saline
ZG	*Zona glomerulosa*
ZF	*Zona fasciculata*
ZR	*Zona reticularis*
A	Adrenaline
NA	Noradrenaline

## References

[1] Vogt M. (1954). The Role of the Adrenal Gland in Homeostasis. Q. J. Exp. Physiol. Cogn. Med. Sci. Transl. Integr..

[2] De Silva D., Wijesiriwardene B. (2007). The Adrenal Glands and Their Functions. Ceylon Med. J..

[3] Peters A., Conrad M., Hubold C., Schweiger U., Fischer B., Fehm H.L. (2007). The Principle of Homeostasis in the Hypothalamus-Pituitary-Adrenal System: New Insight from Positive Feedback. Am. J. Physiol.-Regul. Integr. Comp. Physiol..

[4] Armario A. (2006). The Hypothalamic-Pituitary-Adrenal Axis: What Can It Tell Us about Stressors?. CNS Neurol. Disord.-Drug Targets (Former. Curr. Drug Targets-CNS Neurol. Disord.).

[5] Papadimitriou A., Priftis K.N. (2009). Regulation of the Hypothalamic-Pituitary-Adrenal Axis. Neuroimmunomodulation.

[6] Harvey S., Phillips J.G., Rees A., Hall T.R. (1984). Stress and Adrenal Function. J. Exp. Zool..

[7] Möstl E., Palme R. (2002). Hormones as Indicators of Stress. Domest. Anim. Endocrinol..

[8] Ehrhart-Bornstein M., Bornstein S.R. (2008). Stress Hormones and Immune Function. Elsevier.

[9] Tsigos C., Chrousos G.P. (2002). Hypothalamic–Pituitary–Adrenal Axis, Neuroendocrine Factors and Stress. J. Psychosom. Res..

[10] Medina Santana C., Slattery O., O’Donovan J., Murphy S. (2025). Histological and Proteomic Approaches to Assessing the Adrenal Stress Response in Common Dolphins (*Delphinus delphis*). Animals.

[11] Burgess E.A., Hunt K.E., Kraus S.D., Rolland R.M. (2017). Adrenal Responses of Large Whales: Integrating Fecal Aldosterone as a Complementary Biomarker to Glucocorticoids. Gen. Comp. Endocrinol..

[12] Kuiken T., Höfle U., Bennett P.M., Allchin C.R., Kirkwood J.K., Baker J.R., Appleby E.C., Lockyer C.H., Walton M.J., Sheldrick M.C. (1993). Adrenocortical Hyperplasia, Disease and Chlorinated Hydrocarbons in the Harbour Porpoise (*Phocoena phocoena*). Mar. Pollut. Bull..

[13] St. Aubin D.J., Ridgway S.H., Wells R.S., Rhinehart H. (1996). Dolphin Thyroid and Adrenal Hormones: Circulating Levels in Wild and Semidomesticated Tursiops Truncatus, and Influence of Sex, Age, and Season. Mar. Mamm. Sci..

[14] Avisse C., Marcus C., Patey M., Ladam-Marcus V., Delattre J.-F., Flament J.-B. (2000). Surgical Anatomy and Embryology of the Adrenal Glands. Surg. Clin. N. Am..

[15] Zwemer R.L. (1936). A Study of Adrenal Cortex Morphology. Am. J. Pathol..

[16] Neville A.M., O’hare M.J. (1985). Histopathology of the Human Adrenal Cortex. Clin. Endocrinol. Metab..

[17] Gorgas K., Böck P. (1976). Morphology and Histochemistry of the Adrenal Medulla: I. Various Types of Primary Catecholamine-Storing Cells in the Mouse Adrenal Medulla. Histochemistry.

[18] Siasios A., Delis G., Tsingotjidou A., Pourlis A., Grivas I. (2022). Adrenal Glands of Mice and Rats: A Comparative Morphometric Study. Lab. Anim..

[19] Milano E.G., Basari F., Chimenti C. (1997). Adrenocortical and Adrenomedullary Homologs in Eight Species of Adult and Developing Teleosts: Morphology, Histology, and Immunohistochemistry. Gen. Comp. Endocrinol..

[20] Barszcz K., Przespolewska H., Olbrych K., Czopowicz M., Klećkowska-Nawrot J., Goździewska-Harłajczuk K., Kupczyńska M. (2016). The Morphology of the Adrenal Gland in the European Bison (*Bison bonasus*). BMC Vet. Res..

[21] Zhongjie L. (1988). The Adrenal Gland of Chinese River Dolphin (*Lipotes vexillifer*). Acta Hydrobiol. Sin..

[22] Bourne G.H. (1949). The Mammalian Adrenal Gland.

[23] Jacobsen A.P. (1941). Endocrinological Studies in the Blue Whale: (Balaenoptera musculus L.).

[24] Vuković S., Lucić H., Živković A., Duras Gomerčić M., Gomerčić T., Galov A. (2010). Histological Structure of the Adrenal Gland of the Bottlenose Dolphin (*Tursiops truncatus*) and the Striped Dolphin (*Stenella coeruleoalba*) from the Adriatic Sea. J. Vet. Med. Ser. C Anat. Histol. Embryol..

[25] Clark L.S., Pfeiffer D.C., Cowan D.F. (2005). Morphology and Histology of the Atlantic Bottlenose Dolphin (*Tursiops truncatus*) Adrenal Gland with Emphasis on the Medulla. J. Vet. Med. Ser. C Anat. Histol. Embryol..

[26] Clark L.S., Cowan D.F., Pfeiffer D.C. (2008). A Morphological and Histological Examination of the Pan-Tropical Spotted Dolphin (*Stenella attenuata*) and the Spinner Dolphin (*Stenella longirostris*) Adrenal Gland. J. Vet. Med. Ser. C Anat. Histol. Embryol..

[27] Carballeira A., Brown J.W., Fishman L.M., Trujillo D., Odell D.K. (1987). The Adrenal Gland of Stranded Whales (*Kogia breviceps* and *Mesoplodon europaeus*): Morphology, Hormonal Contents, and Biosynthesis of Corticosteroids. Gen. Comp. Endocrinol..

[28] Simpson J.G., Gardner M.B. (1972). Comparative Microscopic Anatomy of Selected Marine Mammals. Mamm. Sea Biol. Med..

[29] Clark L.S., Cowan D.F., Pfeiffer D.C. (2006). Morphological Changes in the Atlantic Bottlenose Dolphin (*Tursiops truncatus*) Adrenal Gland Associated with Chronic Stress. J. Comp. Pathol..

[30] Jefferson T.A., Fertl D., Bolaños-Jiménez J., Zerbini A.N. (2009). Distribution of Common Dolphins (*Delphinus* Spp.) in the Western Atlantic Ocean: A Critical Re-Examination. Mar. Biol..

[31] Selzer L.A., Payne P.M. (1988). The Distribution of White-Sided (*Lagenorhynchus acutus*) and Common Dolphins (*Delphinus delphis*) vs. Environmental Features of the Continental Shelf of the Northeastern United States. Mar. Mamm. Sci..

[32] Vuković S., Lucić H., Đuras Gomerčić M., Galov A., Gomerčić T., Ćurković S., Škrtić D., Domitran G., Gomerčić H. (2011). Anatomical and Histological Characteristics of the Pituitary Gland in the Bottlenose Dolphin (*Tursiops truncatus*) from the Adriatic Sea. Vet. Arh..

[33] Alonso-Almorox P., Blanco A., Fiorito C., Gómez-Villamandos J.C., Risalde M.A., Almunia J., Fernández A. (2025). The Orca (*Orcinus orca*) Pituitary Gland: An Anatomical, Immunohistochemical and Ultrastructural Analysis. Front. Neuroanat..

[34] Alonso-Almorox P., Blanco A., Fiorito C., Sierra E., Suárez-Santana C., Consolli F., Arbelo M., Guzmán R.G., Molpeceres-Diego I., Fernández Gómez A. (2025). Dolphin Pituitary Gland: Immunohistochemistry and Ultrastructural Cell Characterization Following a Novel Anatomical Dissection Protocol and Non-Invasive Imaging (MRI). Animals.

[35] Kuiken T., García-Hartmann M. (1991). Proceedings of the First European Cetacean Society Workshop on Cetacean Pathology: Dissection Techniques and Tissue Sampling. Proceedings of the ECS Newsletter.

[36] Geraci J.R., Lounsbury V.J. (2005). Marine Mammals Ashore: A Field Guide for Strandings.

[37] Reynolds J.E., Rommel S.A. (2018). Anatomical Dissection: Thorax and Abdomen. Encyclopedia of Marine Mammals.

[38] Rommel S.A., Lowenstine L.J. (2001). Gross and Microscopic Anatomy. CRC Hanbook of Marine Mammal Medicine.

[39] Jelinek F., Konecny R. (2011). Adrenal Glands of Slaughtered Bulls, Heifers and Cows: A Histological Study. J. Vet. Med. Ser. C Anat. Histol. Embryol..

[40] Cowan D.F. (1966). Observations on the Pilot Whale Globicephala Melaena: Organweight and Growth. Anat. Rec..

[41] Nicolaides N.C., Willenberg H.S., Bornstein S.R., Chrousos G.P. (2023). Adrenal Cortex: Embryonic Development, Anatomy, Histology and Physiology. Endotext [Internet].

[42] Anderson J., Ross A.H.M. (1980). Ectopic Adrenal Tissue in Adults. Postgrad. Med. J..

[43] Schmidt M., Enthoven L., Van Der Mark M., Levine S., De Kloet E.R., Oitzl M.S. (2003). The Postnatal Development of the Hypothalamic–Pituitary–Adrenal Axis in the Mouse. Int. J. Dev. Neurosci..

[44] Fenwick E., Fajdiga P., Howe N. (1978). Functional and Morphological Characterization of Isolated Bovine Adrenal Medullary Cells. J. Cell Biol..

[45] Coupland R.E., Weakley B.S. (1970). Electron Microscopic Observation on the Adrenal Medulla and Extra-Adrenal Chromaffin Tissue of the Postnatal Rabbit. J. Anat..

[46] Coupland R.E. (1965). Electron Microscopic Observations on the Structure of the Rat Adrenal Medulla: I. The Ultrastructure and Organization of Chromaffin Cells in the Normal Adrenal Medulla. J. Anat..

[47] Bornstein S.R., Gonzalez-Hernandez J.A., Ehrhart-Bornstein M., Adler G., Scherbaum W.A. (1994). Intimate Contact of Chromaffin and Cortical Cells within the Human Adrenal Gland Forms the Cellular Basis for Important Intraadrenal Interactions. J. Clin. Endocrinol. Metab..

[48] Bornstein S.R., Ehrhart-Bornstein M., Scherbaum W.A. (1997). Morphological and Functional Studies of the Paracrine Interaction between Cortex and Medulla in the Adrenal Gland. Microsc. Res. Tech..

[49] Mitani F., Suzuki H., Hata J., Ogishima T. (1994). A Novel Cell Layer without Corticosteroid-Synthesizing Enzymes in Rat Adrenal Cortex: Histochemical Detection and Possible Physiological Role. Endocrinology.

[50] Teixeira B., Kramer B. (1993). The Adrenal Gland of the African Buffalo, Syncerus Caffer: A Light and Electron Microscopic Study. Afr. Zool..

[51] Olukole S.G., Adeagbo M.A., Oke B.O. (2016). Histology and Histochemistry of the Adrenal Gland African Giant Rat (*Cricetomys gambianus*, Waterhouse). Int. J. Morphol..

[52] Miura W., Mizunashi K., Kimura N., Koide Y., Noshiro T., Miura Y., Furukawa Y., Nagura H. (2000). Expression of Stanniocalcin in Zona Glomerulosa and Medulla of Normal Human Adrenal Glands, and Some Adrenal Tumors and Cell Lines. J. Pathol. Microbiol. Immunol..

[53] Cetin Y. (1992). Chromogranin A Immunoreactivity and Grimelius’ Argyrophilia. A Correlative Study in Mammalian Endocrine Cells. Anat. Embryol..

[54] Weiss C., Cahill A., Laslop A., Fischer-Colbrie R., Perlman R.L., Winkler H. (1996). Differences in the Composition of Chromaffin Granules in Adrenaline and Noradrenaline Containing Cells of Bovine Adrenal Medulla. Neurosci. Lett..

[55] Carbone E., Borges R., Eiden L.E., García A.G., Hernández-Cruz A. (2019). Chromaffin Cells of the Adrenal Medulla: Physiology, Pharmacology, and Disease. Compr. Physiol..

[56] Paul B., Sarkar S., Islam M.N., Das R. (2016). Morphological and Histological Investigations on the Adrenal Glands in Black Bengal Goat (Capra Hircus). J. Sylhet Agric. Univ..

[57] Abdellatif A.B., Fernandes-Rosa F.L., Boulkroun S., Zennaro M.C. (2022). Vascular and Hormonal Interactions in the Adrenal Gland. Front. Endocrinol..

[58] Gomez-Sanchez C.E. (2007). Regulation of Adrenal Arterial Tone by Adrenocorticotropin: The Plot Thickens. Endocrinology.

[59] Vinson G.P., Pudney J.A., Whitehouse B.J. (1985). The Mammalian Adrenal Circulation and the Relationship between Adrenal Blood Flow and Steroidogenesis. J. Endocrinol..

[60] Breed D., Meyer L.C.R., Steyl J.C.A., Goddard A., Burroughs R., Kohn T.A. (2019). Conserving Wildlife in a Changing World: Understanding Capture Myopathy—A Malignant Outcome of Stress during Capture and Translocation. Conserv. Physiol..

[61] Turner J., Clark L., Haubold E. (2006). Organ Weights and Growth Profiles in Bottlenose Dolphins (*Tursiops truncatus*) from the Northwestern Gulf of Mexico. Aquat. Mamm..

[62] Lalli E. (2010). Adrenal Cortex Ontogenesis. Best Pract. Res. Clin. Endocrinol. Metab..

[63] Venn-Watson S., Colegrove K.M., Litz J., Kinsel M., Terio K., Saliki J., Fire S., Carmichael R., Chevis C., Hatchett W. (2015). Adrenal Gland and Lung Lesions in Gulf of Mexico Common Bottlenose Dolphins (*Tursiops truncatus*) Found Dead Following the Deepwater Horizon Oil Spill. PLoS ONE.

[64] Lair S., Béland P., De Guise S., Martineau D. (1997). Adrenal Hyperplastic and Degenerative Changes in Beluga Whales. J. Wildl. Dis..

[65] Martí O., Gavaldà A., Jolín T., Armario A. (1993). Effect of Regularity of Exposure to Chronic Immobilization Stress on the Circadian Pattern of Pituitary Adrenal Hormones, Growth Hormone, and Thyroid Stimulating Hormone in the Adult Male Rat. Psychoneuroendocrinology.

[66] Ulrich-Lai Y.M., Figueiredo H.F., Ostrander M.M., Choi D.C., Engeland W.C., Herman J.P. (2006). Chronic Stress Induces Adrenal Hyperplasia and Hypertrophy in a Subregion-Specific Manner. Am. J. Physiol. Metab..

[67] Koko V., Djordjeviæ J., Cvijiæ G., Davidoviæ V. (2004). Effect of Acute Heat Stress on Rat Adrenal Glands: A Morphological and Stereological Study. J. Exp. Biol..

[68] Berger I., Werdermann M., Bornstein S.R., Steenblock C. (2019). The Adrenal Gland in Stress—Adaptation on a Cellular Level. J. Steroid Biochem. Mol. Biol..

[69] Spencer K.A. (2017). Developmental Stress and Social Phenotypes: Integrating Neuroendocrine, Behavioural and Evolutionary Perspectives. Philos. Trans. R. Soc. B Biol. Sci..

[70] DeRijk R., De Kloet E.R. (2005). Corticosteroid Receptor Genetic Polymorphisms and Stress Responsivity. Endocrine.

[71] Charmandari E. (2012). Primary Generalized Glucocorticoid Resistance and Hypersensitivity: The End-Organ Involvement in the Stress Response. Sci. Signal..

[72] Kinlein S., Karatsoreos I.N. (2020). The Hypothalamic-Pituitary-Adrenal Axis as a Substrate for Stress Resilience: Interactions with the Circadian Clock. Front. Neuroendocrinol..

[73] Herman J.P., McKlveen J.M., Ghosal S., Kopp B., Wulsin A., Makinson R., Scheimann J., Myers B. (2016). Regulation of the Hypothalamic-pituitary-adrenocortical Stress Response. Compr. Physiol..

[74] Yiallouris A., Filippou C., Themistocleous S.C., Menelaou K., Kalodimou V., Michaeloudes C., Johnson E.O. (2024). Aging of the Adrenal Gland and Its Impact on the Stress Response. Vitam. Horm..

[75] Li N., Li Y., Lu Y., Zhang K., Wang S., Wang C. (2025). The Impact of Chronic Stress on Cortical Thickness in Patients with Depression. Front. Psychiatry.

[76] Peltier H., Jepson P.D., Dabin W., Deaville R., Daniel P., Van Canneyt O., Ridoux V. (2014). The Contribution of Stranding Data to Monitoring and Conservation Strategies for Cetaceans: Developing Spatially Explicit Mortality Indicators for Common Dolphins (*Delphinus delphis*) in the Eastern North-Atlantic. Ecol. Indic..

[77] Coombs E.J., Deaville R., Sabin R.C., Allan L., O’Connell M., Berrow S., Smith B., Brownlow A., Doeschate M.T., Penrose R. (2019). What Can Cetacean Stranding Records Tell Us? A Study of UK and Irish Cetacean Diversity over the Past 100 Years. Mar. Mamm. Sci..

[78] Arbelo M., de Los Monteros A.E., Herráez P., Andrada M., Sierra E., Rodríguez F., Jepson P.D., Fernández A. (2013). Pathology and Causes of Death of Stranded Cetaceans in the Canary Islands (1999–2005). Dis. Aquat. Organ..

[79] Lennon R.L., Storm J., Koger R., Thompson E., Williams R.S., Dagleish M.P., Babayan S.A., ten Doeschate M.T.I., Davison N.J., Brownlow A.C. (2025). The Dead Do Tell Tales: Using Pathology Data From Cetacean Necropsy Reports to Gain Insights Into Animal Health. Ecol. Evol..

[80] Ishimura K., Fujita H. (1997). Light and Electron Microscopic Immunohistochemistry of the Localization of Adrenal Steroidogenic Enzymes. Microsc. Res. Tech..

[81] Kawaoi A. (1969). Ultrastructural Zonation of the Human Adrenal Cortex. Acta Pathol. Jpn..

[82] Mäusle E. (1974). Ultrastructure and Function of the Mesenchyme of the Rat Adrenal Cortex. Beitr. Pathol..

[83] Pihlajoki M., Dörner J., Cochran R.S., Heikinheimo M., Wilson D.B. (2015). Adrenocortical Zonation, Renewal, and Remodeling. Front. Endocrinol..

[84] Carmichael S., Winkler H. (1985). The Adrenal Chromaffin Cell. Sci. Am..

[85] Winkler H. (1976). The Composition of Adrenal Chromaffin Granules: An Assessment of Controversial Results. Neuroscience.

