# Supplementary material for: Morphological, Histological and Ultrastructural Characterization of the Common Dolphin’s Adrenal Glands
Source: Vet Sci. 2026 Apr 2;13(4):348. doi: 10.3390/vetsci13040348 (PMC13120033; doi:10.3390/vetsci13040348)
Supplement: Supplementary file 1 [file vetsci-13-00348-s001.zip › vetsci-4211945-supplementary.pdf]

Supplementary information for:

Morphological, Histological and Ultrastructural Characterization of the Common Dolphin's Adrenal Glands

Paula Alonso-Almorox [1,2,*], Alfonso Blanco [2], Ignacio Molpeceres-Diego [1], Raiden Grandía Guzmán, Diego Llinás Rueda and Antonio Fernández [1,*]

1 Veterinary Histology and Pathology, Atlantic Center for Cetacean Research (CAIC), Institute of Animal Health and Food Safety (IUSA), Veterinary School, University of Las Palmas de Gran Canaria (ULPGC), Trasmontaña s/n, 35413 Arucas, Spain

2 Department of Anatomy and Comparative Pathology and Anatomy, University of Cordoba, 14014 Cordoba, Spain

* Correspondence: paulaalonsoalmx@gmail.com (PAA); antonio.fernandez@ulpgc.es (A.F.)

Table S1. *Complete morphometric and histomorphometric dataset of Delphinus delphis included in the study.*

Sample ID	Sex	Sexual maturity	Body length (cm)	Left adrenal weight (g)	Right adrenal weight (g)	Left adrenal length (cm)	Right adrenal length (cm)	Cx_L_1 (mm)	M_L (mm)	Cx_L_2 (mm)	Cx_R_1 (mm)	M_R (mm)	Cx_R_2 (mm)
i907/16	F	Immature	190	11.52	12.30	5.4	6.5	4	5	3	4	4	4
i129/17	M	Immature	122	1.40	2.00	2.4	2.4	1	5	1	2	4	1
i301/17	M	Immature	151	3.00	3.30	3.5	3.7	1	4	1	1	4	1
i359/17	M	mature	206	7.63	6.84	4.5	5.0	4	3	3	3	5	3
i160/17	F	mature	187	7.00	6.70	4.5	4.0	6	9	3	4	6	3
SA012/18	F	mature	190	ND	ND	5.0	5.6	4	8	3	2	7	5
SA066/18	F	mature	196	ND	ND	4.0	4.0	3	5	1	3	5	1
SA079/19	M	Immature	99	1.00	0.70	1.5	2.1	1	5	1	1	4	1
SA256/18	M	mature	223	7.30	7.16	6.4	6.2	4	3	4	4	2	4
SA075/19	M	Immature	173	2.50	2.60	3.5	3.4	1	4	1	1	4	1
SA332/19	M	mature	197	ND	ND	10.0	8.0	2	6	2	2	4	2
SA082/20	F	mature	195	14.20	10.80	5.6	5.0	5	1	5	ND	ND	ND
SA374/20	F	Immature	134	1.70	1.70	2.3	2.6	1	4	2	1	4	1
SA381/20	M	Immature	136	2.40	3.10	3.1	3.8	2	4	1	2	5	1
SA074/21	M	mature	205	ND	ND	4.9	5.3	2	7	3	2	5	1
SA305/21	M	mature	210	3.70	3.80	3.6	5.5	1	7	1	2	4	2
SA672/21	F	mature	222	ND	ND	6.5	6.2	6	5	3	5	2	4
SA812/21	F	mature	191	8.00	6.00	5.7	5.5	1	4	2	3	5	1
SA244/22	F	mature	104	4.82	5.30	5.2	5.7	4	4	3	9	8	2
SA243/22	F	mature	218	10.00	9.00	5.3	6.0	3	5	3	1	7	4
SA318/22	F	mature	207	6.40	9.20	5.1	4.0	3	3	3	2	3	2
SA334/22	M	mature	198	ND	ND	4.6	5.8	2	4	1	3	4	1
SA373/22	F	mature	204	ND	ND	4.0	3.6	2	4	2	3	5	2
SA395/22	M	mature	219	10.00	11.00	5.4	5.1	3	4	4	3	2	4
SA930/22	M	mature	235	7.80	7.70	5.9	5.8	2	4	2	1	4	2
SA928/22	M	Immature	93	1.40	1.60	2.0	2.2	1	4	1	1	3	1
SA1539/22	M	mature	250	12.40	12.30	5.5	6.0	5	6	4	4	5	3
SA346/23	F	mature	188	6.40	7.20	4.5	5.0	4	5	3	4	4	2
SA362/23	M	Immature	97	0.90	1.00	2.0	2.3	1	3	1	1	4	1
SA463/23	F	Immature	104	2.00	2.00	3.5	3.0	1	2	1	1	3	1
SA312/24	F	mature	190	ND	ND	3.5	4.0	2	5	3	3	4	1
SA364/24	M	mature	210	ND	ND	ND	ND	3	4	5	5	3	4
SA451/24	M	mature	219	7.00	7.90	4.4	5.4	3	7	4	3	4	3
SA481/24	F	mature	193	8.50	9.30	4.3	5.5	ND	ND	ND	ND	ND	ND
SA523/24	F	mature	203	7.90	7.60	5.0	4.5	3	5	4	3	4	3
SA630/24	M	mature	212	7.80	8.20	5.0	5.5	3	4	3	4	4	3
SA1194/24	F	mature	213	8.00	7.00	4.8	4.2	3	15	6	5	18	3
SA240/22	M	mature	194	ND	ND	3.8	4.3	3	2	3	4	2	4
SA452/22	M	mature	211	ND	ND	ND	ND	2	6	2	3	7	2
SA1650/22	M	Immature	192	5.00	5.20	5.0	5.2	3	5	2	3	4	1
SA072/23	F	Immature	186	3.00	4.00	3.5	3.6	2	6	2	1	5	3
SA073/23	F	mature	191	3.00	4.00	3.8	4.1	2	5	2	3	4	1
SA170/23	M	mature	219	6.00	5.00	3.0	4.0	ND	ND	ND	ND	ND	ND
SA217/23	M	mature	210	ND	ND	ND	ND	ND	ND	ND	ND	ND	ND
SA218/23	F	mature	213	ND	ND	ND	ND	ND	ND	ND	ND	ND	ND
SA247/23	F	Immature	186	2.60	3.40	3.4	4.1	1	5	1	1	5	1
SA414/23	M	Immature	201	5.40	4.30	4.4	4.2	2	3	3	1	2	2
SA689/23	F	mature	217	ND	ND	4.0	4.0	2	6	2	1	3	1
SA271/24	M	mature	220	7.00	6.00	4.2	3.9	5	7	3	1	2	4
SA16/25	M	mature	197	6.00	4.00	5.0	4.7	3	4	3	2	5	2
SA17/25	F	mature	190	5.00	4.00	4.3	5.0	2	4	1	ND	ND	ND
SA279/25	F	Immature	187	5.00	6.00	4.5	5.0	1	7	2	2	6	2
SA306/25	M	mature	192	4.00	4.00	3.5	4.2	2	6	2	2	10	2
SA451/25	F	Immature	182	4.00	ND	3.7	ND	ND	ND	ND	ND	ND	ND
SA452/25	F	Immature	186	4.00	5.00	4.3	4.5	1	3	1	1	2	2

Comprehensive dataset of all examined individuals, including sample identification, sex, sexual

maturity status, total body length, absolute adrenal gland weight and length (left and right), and detailed histomorphometric measurements of cortical (Cx_1, Cx_2) and medullary (M) thickness for both glands.
Corticomedullary measurements are expressed in millimetres (mm). Adrenal weights are expressed in grams (g), and adrenal lengths and body length in centimetres (cm).
"ND" indicates measurements not available due to tissue damage, autolysis, incomplete sampling, or technical limitations during processing.This table constitutes the full dataset used for all morphometric and statistical analyses presented in the manuscript.

Sample ID	Sex	Cause of death	Cause of death onset category
i359/17	M	Medical / Degenerative / Organ failure	Non-acute COD
i160/17	F	Parasitic disease	Non-acute COD
SA256/18	M	Fishing Interaction	Acute-COD
SA332/19	M	Medical / Degenerative / Organ failure	Non-acute COD
SA082/20	F	Fishing Interaction	Acute-COD
SA074/21	M	Infectious disease	Non-acute COD
SA305/21	M	Infectious disease	Non-acute COD
SA672/21	F	Infectious disease	Non-acute COD
SA812/21	F	Infectious disease	Non-acute COD
SA244/22	F	Infectious disease	Non-acute COD
SA243/22	F	Infectious disease	Non-acute COD
SA318/22	F	Infectious disease	Non-acute COD
SA930/22	M	Medical / Degenerative / Organ failure	Non-acute COD
SA1539/22	M	Parasitic disease	Non-acute COD
SA346/23	F	Medical / Degenerative / Organ failure	Non-acute COD
SA312/24	F	Infectious disease	Non-acute COD
SA364/24	M	Parasitic disease	Non-acute COD
SA481/24	F	Infectious disease	Non-acute COD
SA523/24	F	Infectious disease	Non-acute COD
SA630/24	M	Infectious disease	Non-acute COD
SA1194/24	F	Infectious disease	Non-acute COD
SA240/22	M	Fishing Interaction	Acute-COD
SA452/22	M	Fishing Interaction	Acute-COD
SA073/23	F	Fishing Interaction	Acute-COD
SA170/23	M	Fishing Interaction	Acute-COD
SA217/23	M	Fishing Interaction	Acute-COD
SA218/23	F	Fishing Interaction	Acute-COD
SA689/23	F	Fishing Interaction	Acute-COD
SA271/24	M	Fishing Interaction	Acute-COD
SA16/25	M	Fishing Interaction	Acute-COD
SA17/25	F	Fishing Interaction	Acute-COD
SA306/25	M	Fishing Interaction	Acute-COD

Table S2. *Sexually mature Delphinus delphis included in cause-of-death comparative analyses.*

This table lists all sexually mature individuals with a clearly assigned cause-of-death (COD) classification that were included in comparative morphometric and corticomedullary ratio analyses. Individuals were categorised as Acute COD when mortality resulted from fishing interaction (bycatch), and as Non-acute COD when death was attributed to infectious disease, parasitic disease, or medical/degenerative organ failure. This subset defines the adult cohort used for statistical comparisons of corticomedullary ratios between acute and non-acute mortality.

Figure S1. Absolute adrenal morphometry in sexually mature Delphinus delphis by sex.

Adrenal morphometry in sexually mature Delphinus delphis by sex<br>Absolute values (mean ± SD)									
Females (n)	Males (n)	Adrenal weight – Left (g) Females	Adrenal weight – Left (g) Males	Adrenal weight – Right (g) Females	Adrenal weight – Right (g) Males	Adrenal length – Left (cm) Females	Adrenal length – Left (cm) Males	Adrenal length – Right (cm) Females	Adrenal length – Right (cm) Males
19	18	7.44 ± 2.85	7.22 ± 2.36	7.17 ± 2.14	6.99 ± 2.70	4.73 ± 0.77	4.99 ± 1.67	4.77 ± 0.82	5.29 ± 1.05

Values are presented as mean ± standard deviation. No body-size standardisation was applied.

Absolute adrenal weight and length measurements in sexually mature (adult) individuals, presented separately for females and males. Values are expressed as mean ± standard deviation. No body-size standardisation was applied. These data demonstrate overlapping morphometric distributions between sexes and no marked lateral asymmetry between left and right glands.

Figure S2. *Comparison of corticomedullary ratio (CM_2) according to sexual maturity in Delphinus delphis.*

Sexual maturity	n	Median CM_2	IQR CM_2
immature	16	0.60	0.34
mature	34	1.04	0.85

Boxplots illustrate the distribution of CM values in sexually immature (n = 16) and sexually mature (n = 34) individuals. Central lines represent the median, boxes indicate the interquartile range (IQR), and whiskers denote the range of observed values. CM values were compared using the Wilcoxon rank-sum test.

Figure S3. *Comparison of corticomedullary ratio (CM) between acute and non-acute cause-of-death (COD) groups in sexually mature Delphinus delphis.*

Acute COD vs Non-acute COD

Cause of death group	n	Median CM_2	IQR CM_2
Acute COD	10	1.02	2.25
Non-acute COD	18	1.27	0.77

Individuals were classified as Acute COD (n = 10) when mortality resulted from fishing interaction, and as Non-acute COD (n = 18) when death was attributed to infectious, parasitic, or medical/degenerative conditions. Boxplots display median, interquartile range (IQR), and observed range. Differences were assessed using the Wilcoxon rank-sum test.

Table S3. *Mean diameter (nm) of mitochondria and lipid droplets in the adrenal cortex of* Delphinus delphis.

Zone	Structure	Juveniles	Adult female	Adult male
ZG	Mitochondria	544.36 ± 17.98	551.43 ± 16.76	552.72 ± 17.86
ZG	Lipid droplets	645.12 ± 26.32	659.44 ± 21.70	653.74 ± 16.37
ZF	Mitochondria	574.90 ± 28.69	579.23 ± 24.60	575.70 ± 21.25
ZF	Lipid droplets	658.80 ± 27.60	678.54 ± 5.94	669.43 ± 10.55
ZR	Mitochondria	573.73 ± 15.78	580.00 ± 12.24	578.80 ± 22.44
ZR	Lipid droplets	650.19 ± 27.35	661.45 ± 17.21	666.35 ± 12.75

Values are expressed as mean ± SD. Measurements were obtained from six independent fields per cortical zone and age–sex group. Data correspond to intra-individual variability within each specimen (juveniles, n = 2; adult female, n = 1; adult male, n = 1). ZG, zona glomerulosa; ZF, zona fasciculata; ZR, zona reticularis.

Table S4. Mean cell diameter (μm) of adrenal medullary cells in *Delphinus delphis.*

Cell Type	Juveniles	Adult female	Adult male
Chromaffin cells type A	15.13 ± 0.65	15.33 ± 0.43	15.37 ± 0.42
Chromaffin cells type NA	15.07 ± 0.42	15.13 ± 0.43	15.14 ± 0.47

Values are expressed as mean ± standard deviation (SD). Juvenile values represent pooled measurements from two individuals (n = 2), whereas adult female and adult male values correspond to single individuals (n = 1 each) and reflect intra-individual variability.
